# Antitumor Activity of Isalpinin from *Paphiopedilum dianthum* on Non-Small Cell Lung Cancer Cell Lines

**DOI:** 10.3390/molecules30132762

**Published:** 2025-06-27

**Authors:** Phisit Pouyfung, Nonthalert Lertnitikul, Hua Bai, Achitphol Chookaew, Varisa Pongrakhananon, Piriya Chonsut, Suwichak Chaisit

**Affiliations:** 1 Department of Occupational Health and Safety, School of Public Health, Walailak University, Nakhon Si Thammarat 80160, Thailand; phisit.po@mail.wu.ac.th; 2 Biomass and Oil Palm Center of Excellence, Walailak University, Nakhon Si Thammarat 80160, Thailand; 3 Department of Pharmacognosy and Pharmaceutical Botany, Faculty of Pharmaceutical Sciences, Chulalongkorn University, Bangkok 10330, Thailand; nonthalert.l@chula.ac.th; 4 Animal Models of Chronic Inflammation-Associated Diseases for Drug Discovery Research Unit, Chulalongkorn University, Bangkok 10330, Thailand; 5 College of Public Health, Kunming Medical University, Kunming 650500, China; baihua@kmmu.edu.cn; 6 Division of Hematopoiesis, Joint Research Center for Human Retrovirus Infection and Graduate School of Medical Sciences, Kumamoto University, Honjo, Chuou-ku, Kumamoto 860-0811, Japan; achitphol.ch@gmail.com; 7 Department of Pharmacology and Physiology, Faculty of Pharmaceutical Sciences, Chulalongkorn University, Bangkok 10330, Thailand; varisa.p@pharm.chula.ac.th; 8 Center of Excellence in Preclinical Toxicity and Efficacy Assessment of Medicines and Chemicals, Chulalongkorn University, Bangkok 10330, Thailand; 9 Department of Applied Thai Traditional Medicine, School of Medicine, Walailak University, Nakhon Si Tammarat 80160, Thailand; piriya.ch@wu.ac.th; 10 Department of Chemical Engineering and Pharmaceutical Chemistry, School of Engineering and Technology, Walailak University, Nakhon Si Thammarat 80160, Thailand

**Keywords:** *Paphiopedilum dianthum*, isalpinin, flavonoid, anti-proliferation, anti-migration, anchorage-independent growth, non-small lung cancer cells

## Abstract

Lung cancer is a leading cause of cancer-related deaths globally, with current treatments having significant limitations, including drug resistance, metastasis, and tumor heterogeneity. This study investigated the anticancer potential of isalpinin, a flavonoid isolated from *Paphiopedilum dianthum*, against non-small cell lung cancer (NSCLC) cell lines A549, H23, and H460. Isalpinin significantly inhibited NSCLC cell viability in a dose- and time-dependent manner; H23 and H460 cells showed greater sensitivity (IC_50_ a ~ 44 μM at 48 h) compared to A549 cells (IC_50_ 82 μM). Isalpinin suppressed proliferation, migration, and anchorage-independent growth, particularly in H23/H460 cells. Mechanistically, it induced apoptosis via increased ROS production and Bcl-2 downregulation, particularly in H23 and H460 cells. In a molecular docking analysis, isalpinin was found to directly bind to the ATP-binding pocket of AKT1, as confirmed by reduced Akt/GSK3β phosphorylation. These results suggest that isalpinin showed a potent multi-target natural compound against NSCLC that disrupts the key hallmarks of malignancy and pro-survival signaling. However, its subtype-specific efficacy warrants further in vivo studies and an investigation of combinatorial therapeutic approaches to elucidate its clinical potential.

## 1. Introduction

Lung cancer remains one of the most significant global health burdens and the leading cause of cancer-related mortality worldwide [1]. Although its incidence and outcomes vary geographically largely due to differences in smoking prevalence, environmental exposures, and healthcare access, the disease continues to account for a substantial proportion of cancer-related deaths [2,3]. Tobacco smoking is the primary risk factor, but other contributors include occupational and environmental carcinogens (e.g., radon, asbestos), genetic predispositions, and chronic pulmonary conditions [4]. Lung cancer is broadly categorized into non-small cell lung cancer (NSCLC) and small cell lung cancer (SCLC), each with distinct pathological features and treatment challenges [5]. NSCLC comprises approximately 85% of all lung cancer cases and includes adenocarcinoma, squamous cell carcinoma, and large cell carcinoma. Although NSCLC tends to progress more slowly than SCLC, it is often less responsive to conventional therapies. In contrast, SCLC is associated with rapid growth and early metastasis and requires aggressive therapeutic approaches [6]. Despite advancements in molecular-targeted therapies and immunotherapy, major limitations such as drug resistance to EGFR tyrosine kinase inhibitors which often arise from secondary mutations like T790M [7], adverse effects, and the influence of the tumor microenvironment continue to impede successful long-term outcomes [8].

The phosphoinositide 3-kinase (PI3K)/AKT pathway plays a pivotal role in the critical signaling cascades involved in lung tumorigenesis. This pathway regulates cellular processes including growth, survival, metabolism, and motility. The hyperactivation of Akt, particularly through phosphorylation at Ser473, is associated with a poor prognosis and therapeutic resistance in NSCLC. One of its downstream targets, glycogen synthase kinase 3 beta (GSK3β), is inactivated by phosphorylation at Ser9, which contributes to tumor progression. Therefore, modulation of this pathway has emerged as a promising strategy for the development of anticancer drugs.

Flavonoids, a diverse group of naturally occurring polyphenols found in plants, have gained substantial attention for their potential in cancer chemoprevention and therapy due to their anti-inflammatory, antioxidant, and antineoplastic activities [9]. These compounds are known to interact with various oncogenic pathways including the PI3K/Akt and MAPK/ERK pathways. For example, eupafolin, derived from *Salvia plebeia*, was reported to inhibit the proliferation, migration, and invasion of NSCLC cells by modulating FAK/PI3K/Akt signaling and downregulating metastasis-associated markers, such as MMP9 and RhoA [10]; kaempferol derivatives and platanoside from *Angelica acutiloba* induce pyroptosis in A549 cells through the NF-κB/NLRP3 inflammasome pathway [11].

**Figure 1:**
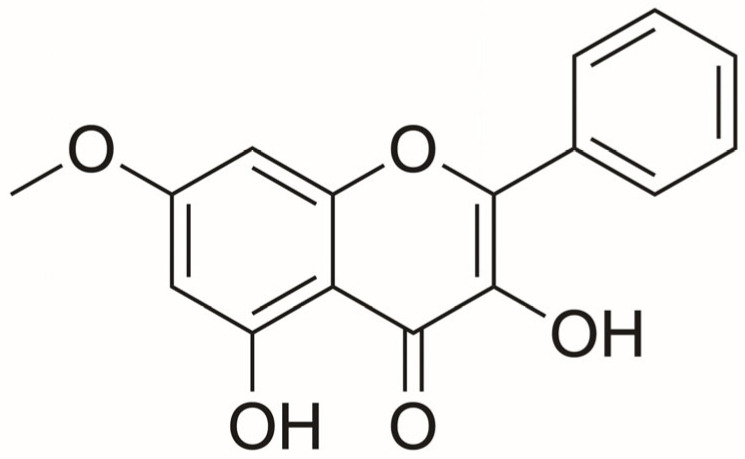
Chemical structure of isalpinin.

Despite the growing body of evidence supporting the anticancer potential of flavonoids, the specific effects and mechanisms of isalpinin, a flavonol isolated from *Paphiopedilum dianthum* (Orchidaceae), in NSCLC remain unexplored. The Orchidaceae family is recognized as a rich source of bioactive compounds with diverse pharmacological activities, including significant anticancer properties. A recent review by [12] highlights that orchid species such as *Dendrobium, Bulbophyllum*, and *Anoectochilus* produce phenanthrenes, bibenzyls, and flavonoids with cytotoxic effects against various cancer cell lines, including lung, breast, and liver cancers. These compounds induce apoptosis, cause cell cycle arrest, and modulate pathways like PI3K/Akt and caspase activation. Notably, dendrobine from *Dendrobium nobile* and compounds from *Anoectochilus roxburghii* trigger apoptosis in tumor cells. The review also underscores the need for further research on the anticancer potential of lesser studied orchid species. *P. dianthum* is an orchid species native to parts of Southeast Asia, including northern Thailand. Phytochemical analyses of its roots have identified isalpinin (Figure 1) as a key constituent, structurally characterized as 5-hydroxy-7-methoxyflavonol based on spectroscopic studies [13,14]. Notably, isalpinin lacks proton signals at C-2 and C-3, supporting its classification as a flavonol. While its effects on non-small cell lung cancer (NSCLC) cell lines have not been previously reported, the primary focus of this study isalpinin has shown selective cytotoxicity against the doxorubicin-resistant breast cancer cell line MCF-7/DOX (IC_50_ = 76.24 ± 1.46 μM), without affecting normal NIH/3T3 fibroblasts [15]. This selectivity may be linked to the presence of a methoxy group at C-7, structurally similar to pinosylvin monomethyl ether, which could influence drug resistance mechanisms. Additionally, isalpinin has been identified as a substrate for breast cancer resistance protein (BCRP), though it does not significantly modulate MRP1 activity [15]. These observations underscore the importance of flavonoid structure activity relationships in determining transporter specificity. Given the global burden of NSCLC and the role of flavonoids in modulating critical oncogenic pathways such as PI3K/Akt, this study aims to investigate isalpinin’s potential as a novel therapeutic agent for NSCLC. This study evaluated anticancer potential of isalpinin in NSCLC by targeting five hallmarks of malignancy: cell survival (cytotoxicity, MTT), proliferation (low-density MTT), metastasis (wound healing), tumorigenicity (soft agar), and apoptosis (Hoechst 33342 staining/Bcl-2 expression). These assays, selected to reflect NSCLC progression mechanisms, collectively demonstrated multi-target activity of isalpinin against survival and metastatic pathways. Mechanistic studies linked its efficacy to PI3K/Akt/GSK3β inhibition, with heightened effects observed in aggressive H23 and H460 subtypes. This supports its potential as a selective therapeutic for advanced NSCLC.

## 2. Results

### 2.1. Cytotoxicity of Isalpinin and Cisplatin on NSCLC Cell Lines

**Figure 2:**
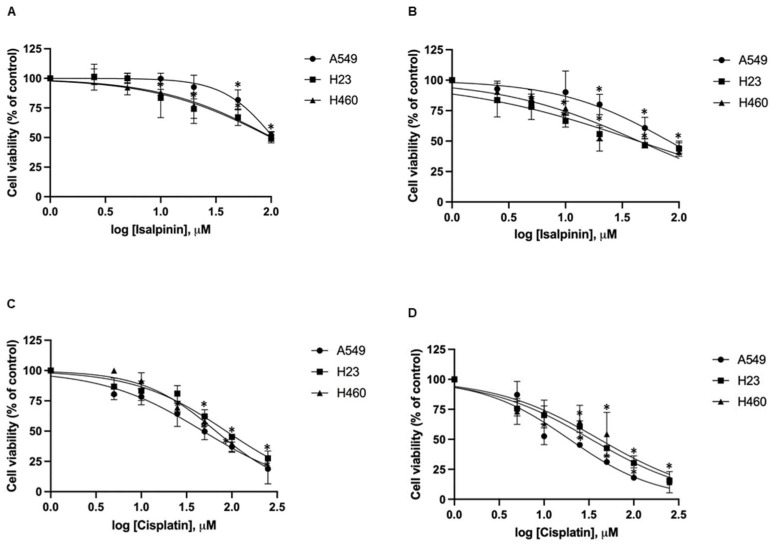
Cytotoxic effects of isalpinin and cisplatin in A549, H23, and H460 lung cancer cells. Cells were treated with various concentrations of isalpinin (0–200 μM) for 24 (**A**) and 48 h. (**B**) or with cisplatin (0–250 μM) for 24 h (**C**) and 48 h (**D**). Cell viability was assessed using the MTT assay and expressed as a percentage relative to the untreated control group. Data are presented as mean ± SEM from three independent experiments (*n* = 3). Dose-response curves were generated by nonlinear regression of log-transformed compound concentrations. * *p* < 0.05, compared with the control group.

To further investigate its anticancer potential, we assessed the cytotoxic potential of isalpinin (0–200 μM) or cisplatin (0–250 μM) for 24 and 48 h. Cell viability was determined using the MTT assay, and IC_50_ values were calculated via nonlinear regression analysis (Figure 2A–D) and * are summarized in Table 1. The results indicate that isalpinin reduced cell viability in a time- and dose-dependent manner across all three NSCLC cell lines. After 48 h of treatment, H23 and H460 cells showed greater sensitivity, with IC_50_ values of 44.34 ± 16.34 μM and 44.46 ± 13.40 μM, respectively, compared to 81.88 ± 23.36 μM in A549 cells. Notably, isalpinin exhibited minimal cytotoxicity toward normal mouse embryonic fibroblast cells (NIH/3T3), with IC_50_ values exceeding 100 μM. In contrast, cisplatin demonstrated significantly higher cytotoxic potency in all tested cell lines, with IC_50_ values of 19.7 ± 3.34 μM (A549), 33.39 ± 2.95 μM (H23), 41.43 ± 8.58 μM (H460), and 14.65 ± 2.58 μM (NIH/3T3). While isalpinin was less potent than cisplatin, its moderate cytotoxicity and preferential activity against H23 and H460 cells suggest promising therapeutic potential, particularly as a lead compound for the development of selective anticancer agents.

### 2.2. Anti-Proliferative Effect of Isalpinin on A549, H23 and H460 NSCLC Cell Lines

**Figure 3:**
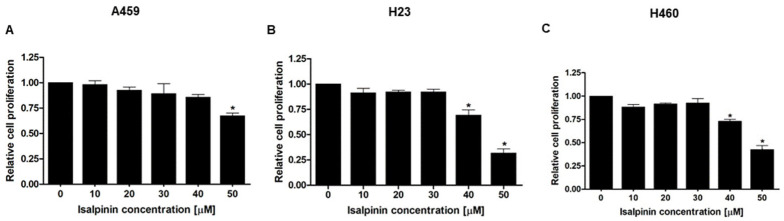
Anti-proliferative effects of isalpinin in NSCLC cell lines. A549 (**A**), H23 (**B**), and H460 (**C**) cells were treated with increasing concentrations of isalpinin (0–50 μM) for 48 h. Cell proliferation was evaluated using the MTT assay, and the results were normalized to the untreated control group. Data are presented as mean ± SEM from three independent experiments (*n* = 3). A concentration- dependent reduction in cell proliferation was observed, with statistically significant inhibition detected in H23 and H460 cells at concentrations ≥ 40 μM and in all three cell lines at 50 μM. * *p* < 0.05 vs. untreated control.

Unregulated cellular proliferation is the hallmark of cancer progression [16]. To evaluate the anti-proliferative potential of isalpinin, A549, H23, and H460 lung cancer cells were treated with increasing concentrations of isalpinin (0–50 μM) for 48 h, and cell proliferation was assessed using the MTT assay. As shown in Figure 3, isalpinin exhibited a dose-dependent inhibitory effect on cell proliferation, with a minimal impact observed at concentrations below 40 μM in A549 cells. In contrast, H23 and H460 cells showed a statistically significant reduction in proliferation at 40 μM, with marked inhibition evident at 50 μM in all the three cell lines. These findings suggest that, while lower concentrations of isalpinin exert minimal cytostatic effects, higher doses significantly reduce cell viability, particularly in H23 and H460 cells, likely through cytotoxic mechanisms.

### 2.3. The Anti-Migratory Effect of Isalpinin on A549, H23, and H460 NSCLC Cell Lines

**Figure 4:**
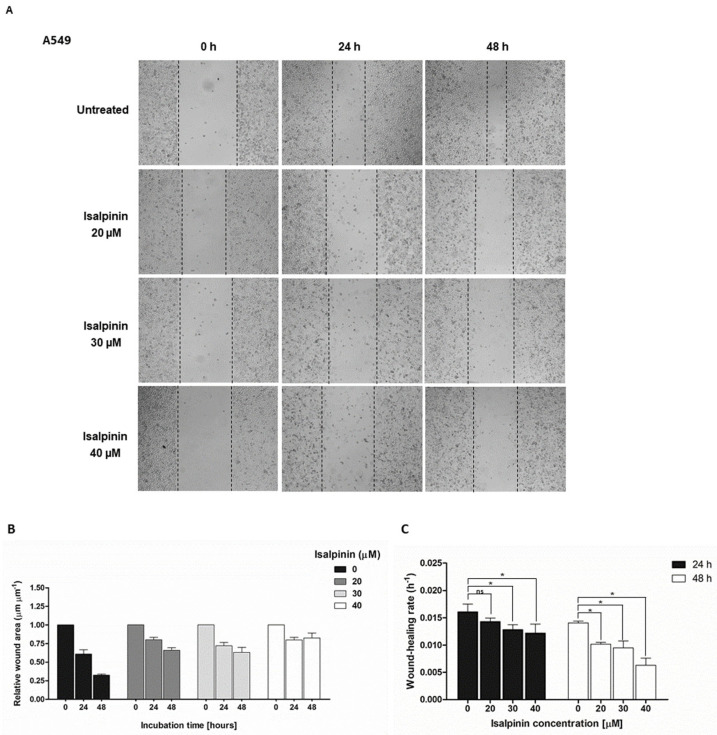

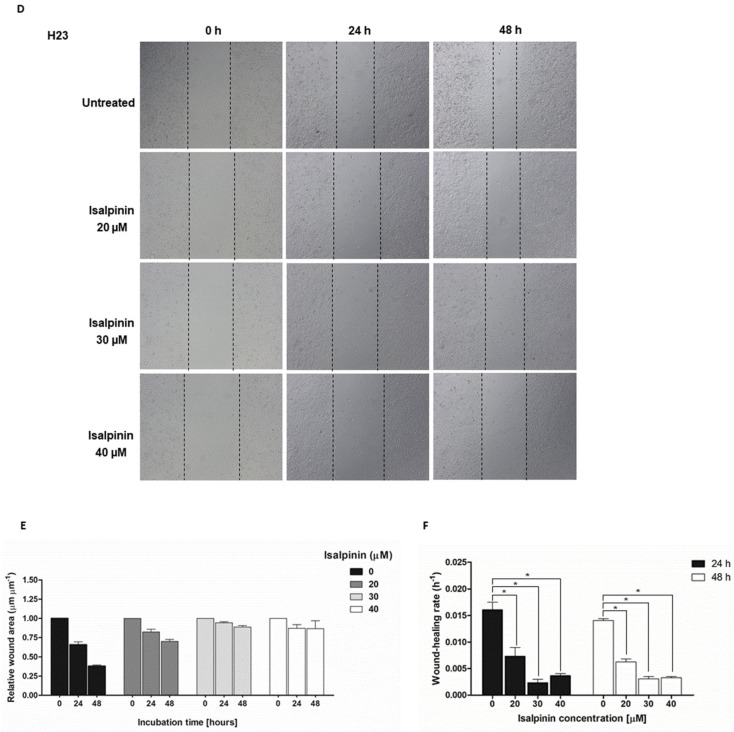

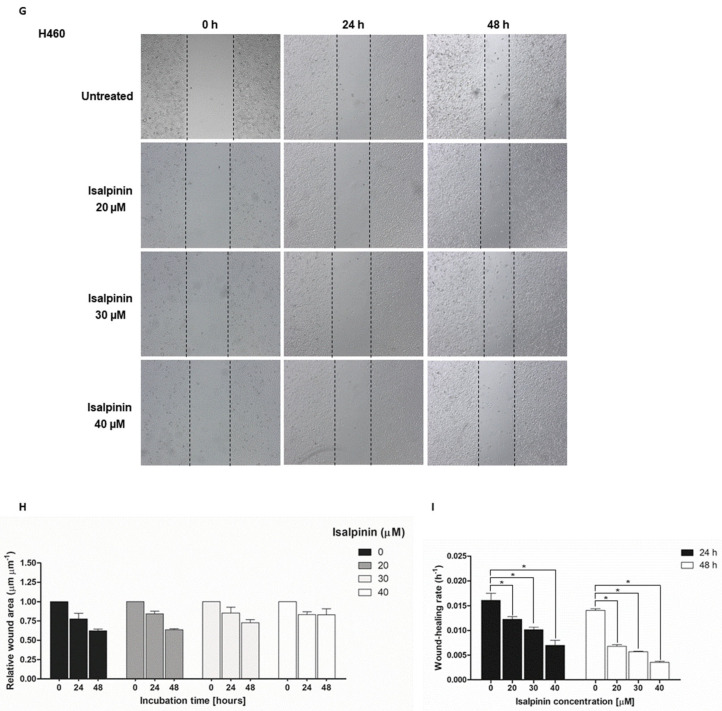
(**A**,**D**,**G**) Representative images of wound closure in A549, H23, and H460 cells treated with isalpinin (0, 20, 30, and 40 µM) for 0, 24, and 48 h. (**B**,**E**,**H**) Quantification of the relative wound area (μm^2^/μm^2^) at each time point demonstrates dose- and time-dependent inhibition of migration by isalpinin. (**C**,**F**,**I**) Wound healing rates (h^−1^) were calculated using linear regression from 0 h to 48 h. Treatment with isalpinin at ≥30 µM significantly reduced wound closure compared to untreated controls in all cell lines. Data represent the mean ± SEM of three independent experiments. * *p* < 0.05, vs. untreated controls.

The anti-migratory effect of isalpinin on A549, H23, and H460 NSCLC cell line migration plays a critical role in cancer metastasis. To evaluate the anti-migratory potential of isalpinin, a scratch wound-healing assay was performed on A549, H23, and H460 NSCLC cell lines. Cells were treated with non-cytotoxic concentrations of isalpinin (0, 20, 30, and 40 µM) to slightly affect proliferation, and wound closure was monitored 24 and 48 h post-scratch (Figure 4A–I). Isalpinin inhibited wound closure in a concentration-dependent manner in all the three cell lines. The result showed that A549 cells (Figure 4A) decreased the relative wound area in untreated control cells; the relative wound area decreased from 1.0 (immediately after scratch, 0 h) to 0.35 at 48 h, corresponding to a wound-healing rate of approximately 0.014 h^−1^. In contrast, treatment with 40 µM isalpinin significantly attenuated wound closure, with the relative wound area decreasing from approximately 1.0 to only 0.75 over the same period. This equated to a wound healing rate of approximately 0.006 h^−1^ (*p* < 0.05), representing an approximate 57% inhibition of cell migration relative to the untreated control (Figure 4B,C). In H23 cells (Figure 4D), the relative wound area in control conditions decreased from approximately 1.0 to 0.35 (healing rate ≈ 0.014 h^−1^) within 48 h. Treatment with 40 µM isalpinin markedly inhibited this process, with the wound area only decreasing from approximately 1.0 to 0.90, yielding a wound healing rate of approximately 0.002 h^−1^. This represented approximately 86% inhibition of migration (Figure 4E,F). Similarly, H460 cells exhibited a reduced migratory capacity following isalpinin treatment. Control H460 cells showed a decrease in wound area from 1.0 to 0.60 (healing rate approximately 0.019 h^−1^) over 48 h, (Figure 4G), whereas in the presence of 40 µM isalpinin, the wound area decreased from 1.0 to 0.80 (healing rate approximately 0.003 h^−1^), corresponding to an approximate 84% inhibition (Figure 4H,I). At all time points and concentrations, H23 cells exhibited the highest sensitivity to isalpinin, followed by H460 and A549 cells. Statistically significant reductions in wound healing were observed in H23 and H460 cells at all concentrations and time points, whereas A549 cells showed significant inhibition only at 40 µM for 24 h. Although the wound-healing assay predominantly assesses cell migration, a potential minor contribution from altered proliferation rates cannot be entirely excluded and warrants further investigation. Collectively, these findings underscore the potential of isalpinin as an inhibitor of metastatic processes in non-small cell lung cancer, primarily through the suppression of cell motility.

### 2.4. Isalpinin Inhibited Anchorage-Independent Growth in A549, H23, and H460 NSCLC Cell Lines

**Figure 5:**
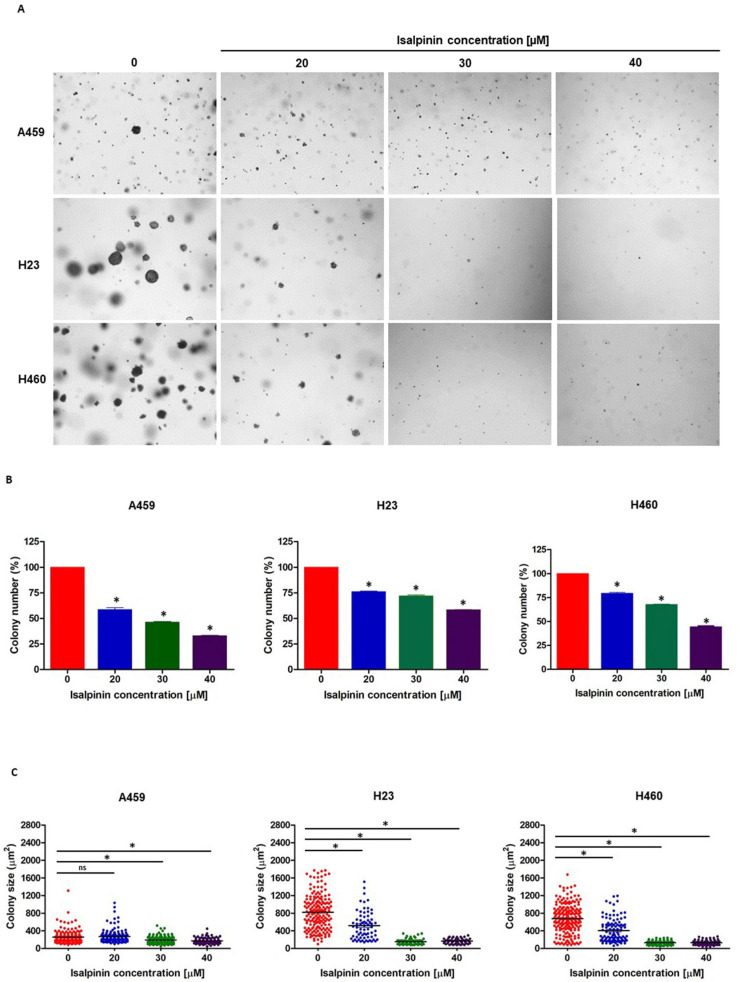
Isalpinin suppresses anchorage-independent growth of NSCLC cell lines. (**A**) Representative images of colony formation in soft agar of A549, H23, and H460 cells treated with isalpinin (0–40 µM) for 14 days. (**B**) Quantification of colony numbers is expressed as percentages relative to the untreated control group. (**C**) Quantification of the colony size (μm^2^) for each treatment group. The data represent the mean ± SEM of three independent experiments. * *p* < 0.05, compared to the untreated control; ns: not significant.

To assess the long-term anti-tumorigenic potential of isalpinin, a soft agar colony formation assay was performed to evaluate its effect on anchorage-independent growth, a hallmark of cellular transformation. A549, H23, and H460 cells were treated with noncytotoxic concentrations of isalpinin (0–40 μM) for 14 days. As shown in Figure 5A, isalpinin treatment markedly reduced colony formation in a dose-dependent manner in all three cell lines. Quantitative analysis demonstrated a significant decrease in colony number at 40 μM isalpinin, with reductions of approximately 67% in A549, 56% in H23, and 42% in H460 cells, respectively, compared to the untreated control (Figure 5B). Similarly, the colony size was significantly reduced by approximately 33%, 84%, and 85% in A549, H23, and H460 cells, respectively (Figure 5C). Notably, in A549 cells, treatment with 20 μM isalpinin did not significantly (ns) alter the colony size, whereas higher concentrations showed clear inhibitory effects (*p* < 0.05). These findings indicate that isalpinin effectively suppresses anchorage-independent growth in lung cancer cells, particularly in H23 and H460 cells, suggesting its potential to impair tumorigenic capacity under nonadherent conditions.

### 2.5. The Effect of Isalpinin on Intracellular Reactive Oxygen Species (ROS) Levels in A549, H23, and H460 NSCLC Cell Lines

**Figure 6:**
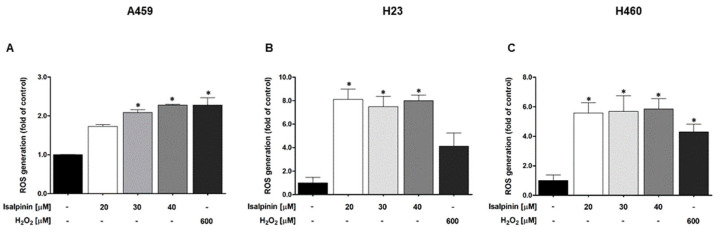
Isalpinin induces intracellular ROS generation in NSCLC cell lines. A549 (**A**), H23 (**B**), and H460 (**C**) cells were treated with isalpinin (20, 30, or 40 μM) or hydrogen peroxide (H_2_O_2_; 600 μM) as a positive control for 1 h. Intracellular reactive oxygen species (ROS) levels were measured using the DCFH_2_-DA fluorescence assay. Data are expressed as fold change relative to untreated control cells and are presented as the mean ± SEM from three independent experiments (*n* = 3). * *p* < 0.05, vs. untreated controls.

The effect of isalpinin on intracellular reactive oxygen species (ROS) levels in A549, H23, and H460 lung cancer cells and elevated levels of intracellular reactive oxygen species (ROS) levels are known to contribute to oxidative stress and may play a role in mediating anticancer activity. To determine whether isalpinin induced ROS generation, A549, H23, and H460 cells were treated with isalpinin at concentrations of 20, 30, and 40 μM for 1 h. Hydrogen peroxide (H_2_O_2_, 600 μM) was used as a positive control. Intracellular ROS levels were quantified using the DCFH_2_-DA fluorescence assay. As shown in Figure 6A–C, isalpinin induced a concentration-dependent increase in ROS production in all cell lines. In A549 cells (Figure 6A), isalpinin at 30 and 40 μM elevated ROS levels by approximately 2-fold relative to the untreated controls. A more pronounced effect was observed in H23 cells (Figure 6B), where ROS levels increased nearly 8-fold at the same concentrations. Similarly, in H460 cells (Figure 6C), isalpinin treatment led to a 6-fold increase in ROS generation. These findings suggest that isalpinin induces significant oxidative stress in lung cancer cells, with H23 and H460 cells showing greater sensitivity to isalpinin-mediated ROS accumulation than the A549 cells. This differential ROS induction might contribute to the cell-specific cytotoxic effects of isalpinin.

### 2.6. Effect of Isalpinin Induces Apoptosis and Modulates Anti-Apoptotic Bcl-2 Protein Expression in NSCLC Cell Lines

**Figure 7:**
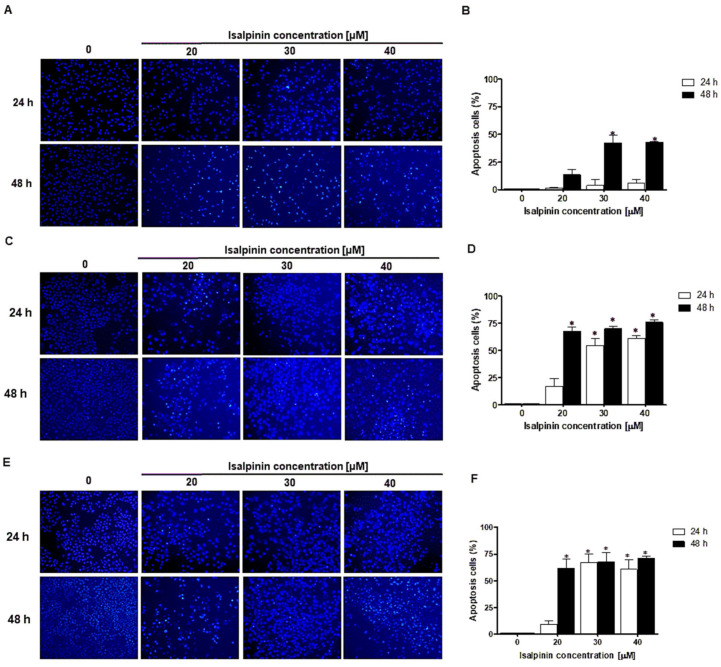

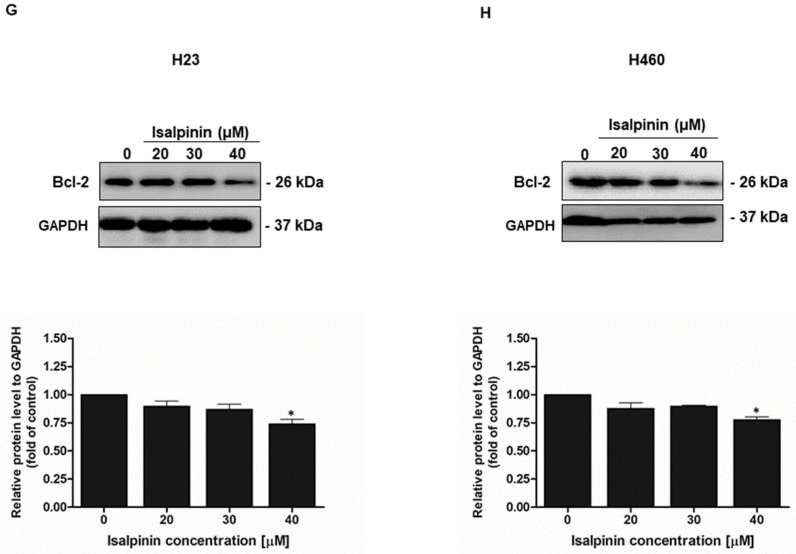
Isalpinin induces apoptosis and downregulates anti-apoptotic protein Bcl-2 in NSCLC cell lines. (**A**,**C**,**E**) Representative fluorescence microscopy images (20× magnification) of Hoechst 33342-stained A549 (**A**), H23 (**C**), and H460 (**E**) cells after treatment with isalpinin (0–40 μM) for 24 and 48 h. Apoptotic cells exhibiting chromatin condensation and nuclear fragmentation are also shown. (**B**,**D**,**F**) Quantification of apoptotic cells in A549 (**B**), H23 (**D**), and H460 (**F**) cell populations, presented as a percentage of total cells. (**G**,**H**) H23 (**G**) and H460 (**H**) cells were treated with isalpinin (0–40 μM) for 24 h, and total protein lysates were analyzed using Western blotting to evaluate Bcl-2 expression levels. GAPDH was used as an internal loading control. Densitometric analysis of Bcl-2 levels relative to GAPDH is shown, expressed as fold change compared to untreated controls. Data are presented as the mean ± SEM from three independent experiments (*n* = 3). * *p* < 0.05 vs. untreated cells.

To investigate whether apoptosis contributes to the anticancer effects of isalpinin, A549, H23, and H460 lung cancer cells were treated with increasing concentrations of isalpinin (0–40 µM) for 24 and 48 h. Apoptotic cell death was assessed using Hoechst 33342 nuclear staining, followed by fluorescence microscopy. As illustrated in Figure 7A–F, isalpinin induced a concentration- and time-dependent increase in apoptosis in all three cell lines. A549 cells (Figure 7A,B) showed a modest apoptotic response, with approximately 6.2% apoptosis at 40 µM after 24 h, but this increased significantly to nearly 50% after 48 h. In contrast, H23 cells (Figure 7C,D) exhibited approximately 60.9% apoptosis at 40 µM after 24 h, with the levels remaining stable after 48 h. H460 cells (Figure 7E,F) demonstrated the highest sensitivity, with approximately 69.7% apoptosis at 40 µM after 24 h, which increased further to approximately 79.8% at 48 h. These findings indicated that isalpinin induces apoptosis in lung cancer cells in a time- and dose-dependent manner, with H460 cells being the most susceptible, followed by H23 and A549 cells.

To further elucidate the apoptotic mechanism induced by isalpinin, the expression levels of the antiapoptotic protein Bcl-2 were examined in H23 and H460 lung cancer cells after a 24 h treatment with isalpinin at concentrations of 0, 20, 30, and 40 μM. As shown in Figure 7G,H, a Western blot analysis revealed a concentration-dependent reduction in Bcl-2 protein levels relative to the GAPDH control. Notably, treatment with 40 μM isalpinin resulted in a significant decrease in Bcl-2 expression by approximately 26% in H23 cells (Figure 7G) and 22% in H460 cells (Figure 7H), compared to the untreated control group (* *p* < 0.05). These findings suggest that isalpinin may promote apoptosis in lung cancer cells, at least in part, through a downregulation of Bcl-2.

### 2.7. Gene Ontology (GO) and KEGG Pathway Enrichment Analysis of Isalpinin-Associated Targets

**Figure 8:**
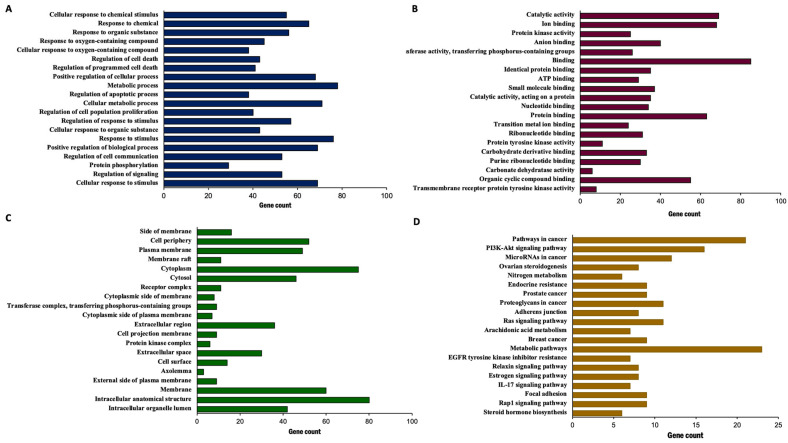
Gene Ontology (GO) and KEGG pathway enrichment analysis of isalpinin-targeted genes. (**A**) Biological Process (BP): Top enriched GO terms related to cell death, chemical response, the regulation of signaling, and apoptosis. (**B**) Molecular Function (MF): enrichment in catalytic activity, ATP binding, protein kinase activity, and nucleotide binding. (**C**) Cellular Component (CC): Targeted proteins are predominantly associated with intracellular anatomical structures, membranes, and cytoplasmic components. (**D**) KEGG Pathway Analysis: Major enriched pathways include cancer-related signaling pathways, such as PI3K/Akt, pathways in cancer, microRNAs in cancer, EGFR-TKI resistance, and hormone-related signaling. Gene counts reflected the number of target genes associated with each GO term or KEGG pathway. Data were analyzed using enrichment tools with a significance threshold of *p* < 0.05.

To elucidate the potential molecular mechanisms underlying the effects of isalpinin in non-small cell lung cancer (NSCLC), gene ontology (GO) framework, and Kyoto Encyclopedia of Genes and Genomes (KEGG) pathway enrichment, analyses were performed on the predicted molecular targets of isalpinin. GO terms were categorized into biological processes (BP), molecular functions (MF), and cellular components (CC). Analysis of the biological processes (Figure 8A), ranked by gene count, indicated significant enrichment in several key areas. These include the regulation of protein phosphorylation, the cellular response to organic substances, the regulation of hormone levels, the response to hormones, the regulation of response to stimuli, protein phosphorylation itself, the regulation of metabolic processes, cellular protein localization, the positive regulation of molecular function (Figure 8B), and the regulation of signaling pathways. In terms of molecular functions, the predicted targets of isalpinin were predominantly associated with transferase activity, particularly involving phosphorus-containing groups, and catalytic activity. Other significantly enriched molecular functions included protein kinase activity, ATP binding, ion binding, enzyme binding, hormone receptor binding, transcription factor binding, transcription corepressor activity, and steroid hormone receptor activity. These findings suggested that isalpinin may mediate its effects by modulating a range of enzymatic and binding activities that are crucial for cellular functions in NSCLC. An analysis of the cellular components revealed that the predicted targets of isalpinin were distributed across various subcellular locations. The enriched terms included membrane components (Figure 8C), cytoplasm, plasma membrane, extracellular region, cytoskeleton, organelles, cytoplasmic vesicles, extracellular space, cell junctions and intracellular anatomical structures. This broad distribution suggests that the molecular targets of isalpinin are located in diverse cellular compartments, potentially affecting multiple cellular pathways and functions. Furthermore, KEGG pathway analysis (Figure 8D) identified a significant enrichment of the predicted targets of isalpinin in pathways that are critically involved in oncogenesis. Notably, these included the PI3K/Akt signaling pathway, pathways related to miRNAs in cancer, general cancer pathways, nitrogen metabolism, endocrine resistance, proteoglycans in cancer, prostaglandins in cancer, the Ras signaling pathway, arachidonic acid metabolism, and pathways associated with EGFR tyrosine kinase inhibitor resistance. The prominent enrichment in the PI3K/Akt pathway and other cancer-associated signaling cascades suggests that isalpinin exerts its anticancer effects by modulating these pivotal oncogenic signaling networks in NSCLC. Collectively, these enrichment analyses provided valuable insights into the potential molecular landscape influenced by isalpinin in NSCLC cells. These findings highlight the potential of isalpinin to target multiple cancer-related pathways and processes, thereby inhibiting cell migration and exerting broader anticancer effects.

### 2.8. Construction of the Protein–Protein Interaction (PPI) Network and Computational Modeling of AKT with Molecular Docking

**Figure 9:**
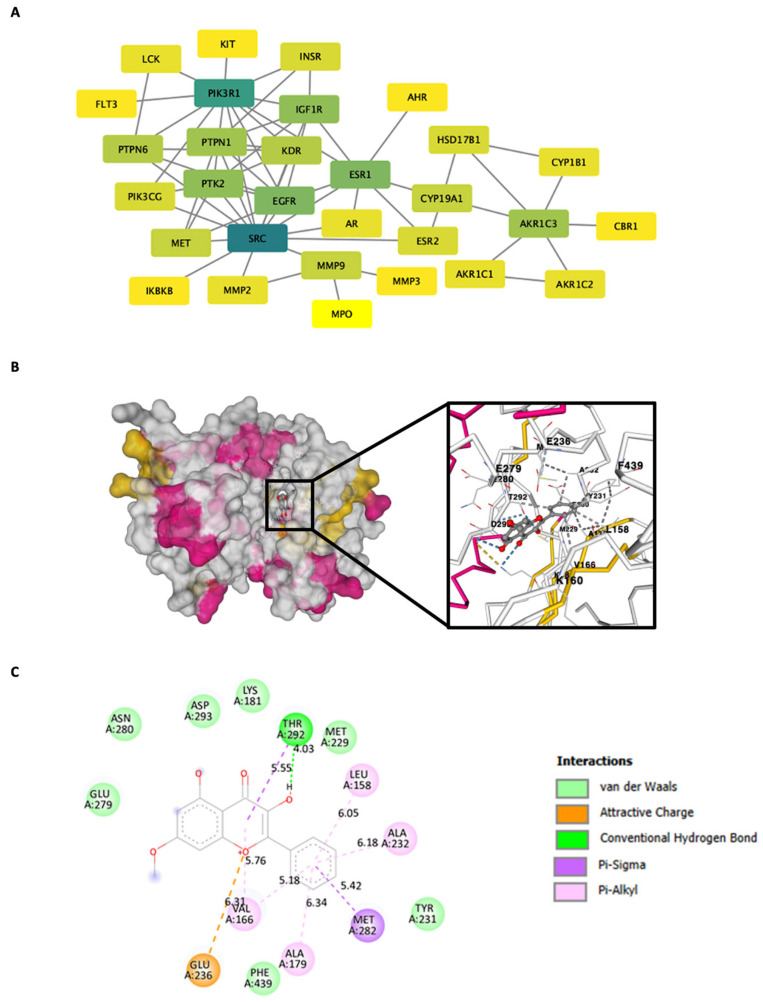
Construction of a protein–protein interaction (PPI) network and molecular docking analysis of isalpinin with AKT1. (**A**) The PPI network of predicted isalpinin target proteins was constructed using the STRING v11.0 database and visualized using Cytoscape. Nodes represent proteins, and edges indicate predicted protein–protein interactions with a confidence score ≥ 0.95. AKT1 (highlighted in dark green) emerged as a central hub, indicating its potential importance in isalpinin-mediated signaling modulation. (**B**) Molecular docking of isalpinin into the active site of AKT1 (PDB ID: 1O6L) using AutoDock Vina on the CB-Dock2 platform. The surface representation illustrates the binding pocket, with the inset highlighting key interactions between isalpinin and the amino acid residues of AKT1 at atomic resolution. The residues involved in binding include Glu236, Ala232, Phe439, and Thr292. (**C**) Two-dimensional interaction map of isalpinin docked into AKT1, generated using the BIOVIA Discovery Studio Visualizer. The ligand interacted with key residues through hydrogen bonds (green lines), π-alkyl interactions (purple), van der Waals forces (light green), and electrostatic contacts (orange). These interactions collectively support the stable binding of isalpinin to AKT1’s ATP-binding site.

To further elucidate the molecular interactions underlying the effect of isalpinin, we constructed a protein–protein interaction (PPI) network to identify central nodes and key pathways potentially modulated by isalpinin in NSCLC cells, and molecular docking studies were conducted focusing on AKT, a central regulator in cancer cell survival and proliferation. A protein–protein interaction (PPI) network was constructed to systematically elucidate the molecular mechanisms by which isalpinin exerts its anticancer effects in NSCLC. This network was built by mapping 43 predicted cancer-related targets using the STRING v11.0 database (Figure 9A), which allowed identification of central nodes and key pathways potentially modulated by isalpinin in NSCLC cells. The PPI network revealed that AKT1 occupies a central node, indicating a high degree of connectivity with other signaling proteins, such as EGFR, PI3KR1, and MAPK1. A topological analysis using Cytoscape prioritized AKT1 based on degree centrality and betweenness metrics, suggesting that it is a key hub protein in the isalpinin–target interaction network. The molecular mechanisms underlying the anti-NSCLC effects of isalpinin, identified through PPI network analysis, were further elucidated through molecular docking simulations. These simulations investigated potential binding interactions between isalpinin and key target proteins. Based on PPI network construction, which identified AKT1 as a central hub protein in the isalpinin–NSCLC interaction network, we focused on characterizing the binding mechanism of isalpinin with AKT1. Docking simulations were conducted using the X-ray crystallographic structure of activated AKT (PDB ID: 1O6L) to predict the binding affinity and interaction sites of isalpinin (CB-Dock2 with AutoDock Vina). The best binding pose (highest negative Vina score) is shown in Figure 9B, illustrating that isalpinin fits into the ATP-binding pocket of AKT1 and interacts with residues critical for kinase function. Detailed interaction profiling (Figure 9C) revealed multiple stabilizing interactions between isalpinin and AKT1. Notably, conventional hydrogen bonding occurred with Glu236 and Thr292, whereas π-alkyl interactions were observed with Phe439, Ala232, and Tyr231, enhancing the affinity of the compound. Additional van der Waals interactions with residues such as Ala166, Met282, and Leu158 further stabilize the ligand–protein complex. These findings suggest that isalpinin may exert anticancer effects, at least in part, by targeting AKT1 through direct binding to its catalytic domain, thereby interfering with downstream pro-survival signaling pathways.

### 2.9. Effect of Isalpinin on Akt/GSK3β Pathway in NSCLC Cell Lines

**Figure 10:**
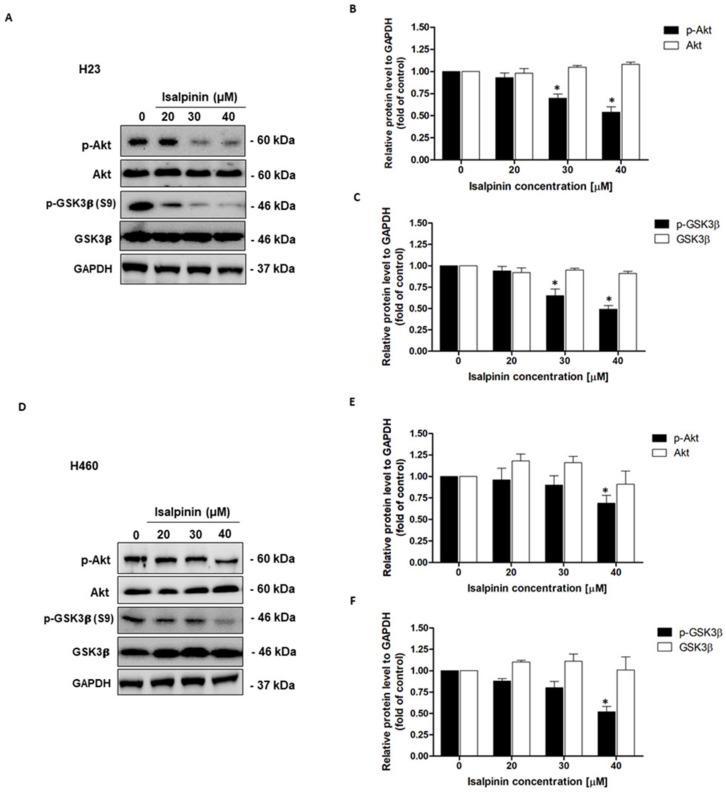
Isalpinin inhibits the Akt/GSK3β signaling pathway in H23 and H460 non-small cell lung cancer (NSCLC) cells. (**A**) Representative Western blot images showing the expression of total Akt, phosphorylated Akt (p-Akt), total GSK3β, and phosphorylated GSK3β (p-GSK3β) in H23 cells treated with 0, 20, 30, and 40 µM isalpinin for 24 h. GAPDH was used as a loading control. (**B**,**C**) Densitometric quantification of p-Akt and p-GSK3β in H23 cells normalized to GAPDH. (**D**) Western blot analysis of the same proteins in H460 cells under identical conditions. (**E**,**F**) Densitometric analysis of p-Akt and p-GSK3β in H460 cells. Data are presented as the mean ± SEM from three independent experiments. * *p* < 0.05, compared to the untreated control group.

Following previous analyses of gene pathways and functional enrichment, which highlighted the significant involvement of the Akt signaling pathway in the action mechanism of isalpinin, further studies were undertaken to determine the direct impact of isalpinin on this pathway in NSCLC cell lines. To assess the expression and phosphorylation levels of crucial proteins in the Akt/GSK3β pathway, H23 and H460 cell lines were treated with increasing concentrations of isalpinin (0, 20, 30, and 40 µM), and the expression levels of Akt, GSK3β, and their phosphorylated forms were evaluated via Western blot analysis. In H23 cells (Figure 10A), treatment with isalpinin led to a dose-dependent decrease in phosphorylated Akt (Ser473) levels, while total Akt levels remained stable. Quantitative densitometry showed that treatment with 30 and 40 µM isalpinin significantly reduced p-Akt levels to approximately 73% and 54%, respectively, compared to untreated control cells, (*p* < 0.05; Figure 10B). Concordantly, the phosphorylation of GSK3β at Serine 9 (p-GSK3β), a downstream target of Akt, was progressively attenuated with increasing concentrations of isalpinin. Treatment with 30 and 40 µM of isalpinin reduced p-GSK3β levels by approximately 68% and 49%, respectively, compared to the control, (*p* < 0.05; Figure 10C), whereas the total GSK3β expression remained constant. A similar inhibitory pattern was observed in H460 cells (Figure 10D). Treatment with 40 µM of isalpinin significantly decreased p-Akt levels to approximately 69% of control values (*p* < 0.05; Figure 10E), with total Akt levels remaining unaffected. Correspondingly, the phosphorylation of GSK3β (Ser9) was also inhibited in a dose-dependent manner, exhibiting a significant reduction to approximately 52% of control levels at 40 µM of isalpinin (*p* < 0.05), without altering total GSK3β protein expression (Figure 10F). These results clearly demonstrate that isalpinin downregulates Akt activation and GSK3β phosphorylation in a concentration-dependent manner in both the H23 and H460 cell lines. The observed suppression of the Akt/GSK3β signaling axis is consistent with the pathway-level predictions from the KEGG enrichment analysis, and provides mechanistic support for the anti-migratory and anti-proliferative effects of isalpinin. These findings underscore the therapeutic potential of isalpinin as a modulator of dysregulated oncogenic signaling in NSCLC.

## 3. Discussion

Cell lung carcinoma is the most common type of lung cancer, and metastasis and apoptosis dysregulation are major obstacles that contribute to its poor clinical outcome [17]. Therefore, therapeutic approaches remain a challenge for anticancer drug discovery. Epidemiological evidence has linked fruit and vegetable consumption to a reduced cancer risk, which is often attributed to bioactive flavonoids. These polyphenols have been shown to exhibit diverse anticancer effects in preclinical models. Natural compounds from orchid species have been shown to possess anticancer properties because they contain up to one hundred types of biologically active flavonols [13,15]. Isalpinin, a methylated flavonol structurally related to common dietary flavonoids, has potential advantages, possibly including enhanced stability or target interactions owing to its methyl group. However, its efficacy, particularly in lung carcinoma, remains unclear. This study aimed to characterize the multifaceted anticancer properties (cancer cell viability, proliferation, mitosis, and apoptosis) of isalpinin, a methylated flavonol isolated from *P. dianthum*, against NSCLC cell lines, including A549, H23, and H460.

Isalpinin exhibited a concentration- and time-dependent inhibition of cell viability across all tested NSCLC cell lines (A549, H23, and H460), as determined by MTT assays. An initial assessment after 24 h of treatment revealed comparable sensitivity across the tested lines, with calculated half-maximal inhibitory concentrations (IC_50_) of 105.2 ± 14.46 µM for A549, 103.1 ± 29.42 µM for H460, and 100.3 ± 24.39 µM for H23 cells. These initial potencies are consistent with previous reports for the non-methylated flavonol quercetin, which exhibited IC_50_ values of 106.3 µM and 92.2 µM against A549 and H460 cells, respectively, after 24 h [18]. Previous research demonstrated that isalpinin exhibited notable cytotoxic activity against the MCF-7 breast cancer cell line, with an IC_50_ value of 52.2  ±  5.9 μM [15], underscoring its broad-spectrum anticancer potential across diverse malignancies. Although its cytotoxic potency at this early time point was initially lower than that of the conventional chemotherapeutic agent, cisplatin, prolonged exposure (48 h) resulted in significantly enhanced cytotoxicity, particularly in H23 and H460 cells. Indeed, extending the exposure duration to 48 h increases IC_50_ values to 44.46 ± 13.40 µM and 44.34 ± 16.34 µM against H460 and H23 cells, respectively. In contrast, A549 cells displayed reduced sensitivity over the same period, with an IC_50_ value of 81.88 ± 23.36 µM. This delayed yet sustained cytotoxic response observed, especially in H23 and H460 cells, may be attributed to the gradual induction of intracellular stress and the engagement of apoptotic pathways. These findings underscore the differential cytotoxic potential of isalpinin, suggesting that its anti-proliferative efficacy may be more pronounced in H460 and H23 cells. The observed IC_50_ values at 48 h confirm a higher susceptibility of H23 and H460 cells to isalpinin-induced cytotoxicity compared to A549 cells, indicating cell line-specific responses potentially due to differences in genetic background, redox balance, or drug transporter expression, in addition to variations in underlying signaling pathway activation [9]. According to the IC_50_ of isalpinin, approximately 40 µM, which may be considered high for direct therapeutic applications, strategies to enhance its clinical translatability are crucial. For further applications, encapsulating isalpinin within nanocarrier systems, such as liposomes, polymeric nanoparticles, or micelles, is a promising solution. Such nanoformulations can significantly improve the pharmacokinetic profile of isalpinin by increasing its solubility, protecting it from premature degradation, and extending its circulation half-life. This approach is highly relevant, as flavonoids are being actively investigated as adjuvants in clinical trials, such as NCT05724329 (dasatinib and quercetin). By utilizing targeted delivery systems to achieve a higher local concentration at the tumor site, isalpinin can be effectively used in synergistic combinations with other anticancer agents, unlocking its full therapeutic potential. Consistent with previous reports on kaempferol bioactivity, kaempferol showed time-dependent cytotoxicity, reaching IC_50_ values of 87.3 µM (A549) and 43.7 µM (H460) after 72 h [19], while kaemferide (Kaempferol 4′-*O*-methyl ether) showed IC_50_ values of 22.5 ± 1.35, 26.2 ± 1.4, and 29.1 ± 1.5 µM against A549, H23, and H460 cell proliferation (24 h incubation time), respectively [20]. Moreover, quercetin (non-methyled flavonol) demonstrated inhibitory effects on A549 human lung carcinoma cells, with an IC_50_ of 72.2 ± 2.3 µM following a 48 h incubation period, whereas isorhamnetin (3′-*O*-methylquercetin) demonstrated a 2.7-fold increase in potency (IC_50_ = 26.6 ± 1.7 µM) relative to quercetin, while tamarixetin (4′-*O*-methylquercetin) showed a 3.7-fold increase (IC_50_ = 19.6 µM) in A549 cells. This enhanced efficacy is linked to structural methylation [21]. Flavonoid methylation is known to influence pharmacokinetic properties, potentially enhancing bioavailability and metabolic stability compared to non-methylated analogs such as quercetin and kaempferol. This hypothesis is supported by the finding that other methylated flavonoids, such as tangeretin and nobiletin, exhibit significant anti-proliferative activity [22]. The notably improved activity of the O-methylated flavonol isorhamnetin suggests that its potential utility as an anticancer agent for non-small cell lung cancer warrants further investigation, as cell migration is a fundamental aspect of cancer metastasis, and inhibition of this process represents a key therapeutic strategy. In this study, we investigated the anti-migratory potential of isalpinin, a methylated flavonol, against lung cancer progression using wound-healing assays in A549, H23, and H460 cell lines. Our findings demonstrated that isalpinin significantly attenuated cell migration in a dose-dependent manner in all three cell lines. Notably, A549 cells exhibited the lowest sensitivity, with wound closure inhibited by approximately 60% after 48 h of treatment with 40 µM isalpinin, whereas significant inhibition was observed in H23 and H460 cells. The observed anti-migratory effects were consistent with previous reports of related flavonols. Myricetin inhibits migration through MMP and EMT pathways [23]. Kaempferol suppresses TGF-β1-induced migration/EMT in A549 cells via the PI3K/Akt1 pathway [24]. Furthermore, methylated flavonol kaempferide reduced A549 cell migration by regulating ROS-mediated TGF-β signaling, decreasing pro-migratory proteins (e.g., SNAI 1, N-cadherin), and increasing E-cadherin expression [20]. These examples support the role of flavonols in targeting key cell migration and EMT pathways. The wound-healing assay revealed that isalpinin potently inhibited NSCLC cell migration in a dose-dependent manner (Figure 4). While transwell invasion assays and EMT marker quantification would provide additional mechanistic insights, the observed two-dimensional migration suppression strongly suggests an anti-metastatic potential. Migration and invasion share overlapping molecular drivers, including PI3K/Akt signaling, which was effectively inhibited in our study (Figure 8 and Figure 9). Akt suppression downregulates MMP-9 and upregulates E-cadherin, thereby counteracting EMT and invasion. Future studies will explore the effects of isalpinin on three-dimensional invasion and EMT markers, such as Snail, Twist, and N-cadherin, to fully elucidate its antimetastatic mechanisms. The ability of isalpinin to interfere with these critical cellular processes underscores its potential therapeutic applications. Subsequent research encompassing detailed mechanistic elucidation and preclinical in vivo efficacy evaluations is imperative for advancing isalpinin for potential clinical applications in lung cancer treatment and the demonstrated significant dose-dependent inhibition of anchorage-independent growth in A549, H23, and H460 (NSCLC) cell lines, as assessed via a 14 day soft agar colony formation assay. At concentrations ranging from 20 µM to 40 µM, isalpinin reduced both the number and size of colonies across all three lines, although with differential sensitivities. H460 cells exhibited the highest sensitivity, followed by H23 cells, whereas A549 cells displayed moderate sensitivity to isalpinin treatment under these conditions. Compared with previous reports, quercetin inhibited A549 colony formation by 27%, 49%, and 83% at concentrations of 25, 50, and 100 μmol/L, respectively, relative to untreated controls. This effect was mechanistically associated with the direct inhibition of Aurora B kinase, a target highly expressed in these cells, as confirmed by reduced sensitivity following Aurora B knockdown [25]. In contrast, the O-methylated quercetin metabolite isorhamnetin exhibited substantially greater potency than quercetin, significantly suppressing A549 colony formation by approximately 32%, 65%, and 82% at markedly lower concentrations of 2.5, 5, and 10 μM, respectively [26]. The inhibitory action of isorhamnetin involves distinct mechanisms, primarily the induction of mitochondrial-dependent apoptosis and the modulation of autophagy, where autophagy inhibition enhances apoptotic cell death [26]. These flavonoids share several common mechanisms in inhibiting colony formation, including the induction of apoptosis through the modulation of pro-apoptotic (Bax and Bad) and anti-apoptotic (Bcl-2) proteins [27,28,29,30], the activation of caspase-dependent pathways leading to cell death [27,28,30], the induction of autophagy, which contributes to cancer cell death [28,29], the inhibition of signaling pathways critical for cancer cell survival and proliferation, particularly PI3K/Akt/mTOR [26], and cell cycle arrest at various phases, preventing cancer cell division and proliferation [27,28,30]. Further research specifically addressing the effects of these natural compounds on H460 and H23 cell colony formation would expand our understanding of their potential as therapeutic agents in lung cancer.

A549, H23, and H460 cells have been reported to exhibit a differential expression of Nrf2/HO-1, a key component of the antioxidant defense system, contributing to distinct redox homeostasis mechanisms [31]. Therefore, we determined whether isalpinin, a methylated flavonol, exerted anticancer effects against ROS generation. A549, H23, and H460 cells were treated with isalpinin (20–40 µM) and intracellular ROS levels were quantified. Isalpinin elevated ROS levels in a concentration-dependent manner in all cell lines. Significant differential sensitivity was observed in H23 cells which showed the largest response (approximately 8-fold ROS increase vs. control), followed by H460 cells (approximately 6-fold), whereas A549 cells exhibited a modest (approximately 2-fold) increase. This sensitivity pattern contrasts sharply with that of kaempferide, which potently induces ROS in A549 cells compared to that in H460 and H23 cells (reportedly via mitochondrial electron transport chain disruption) [20]. Isalpinin showed lower efficacy in A549 cells but higher activity in H23 and H460 cells. The reduced sensitivity of A549 cells to isalpinin may be correlated with their characteristically high expression of Nrf2/HO-1, a key component of the antioxidant defense system known to confer resistance to oxidative stress (e.g., cisplatin resistance) [31]. Conversely, H23 cells exhibited defective Nrf2 activation, and both H23 and H460 cells had lower basal Nrf2/HO-1 levels, potentially rendering them less capable of mitigating isalpinin-induced ROS. While kaempferide effectively overcomes A549 defenses, possibly through direct mitochondrial targeting, isalpinin-induced ROS production appears to be more readily counteracted by the robust Nrf2/HO-1 system in A549 cells [20,31]. Consistent with ROS levels, isalpinin-induced apoptosis was significantly higher and occurred earlier in H23 and H460 cells than in A549 cells. H460 and H23 cells displayed substantial, concentration-dependent apoptosis after 24 h (reaching approximately 60–70% at 40 µM in H460), which increased further by 48 h. In contrast, A549 cells showed minimal apoptosis at 24 h, with significant induction only apparent after 48 h (reaching approximately 50% apoptosis at 40 µM). This direct correlation between the magnitude of ROS generation and apoptotic rates underscores the role of oxidative stress in the cytotoxicity of isalpinin and highlights the influence of cellular antioxidant capacity modulated by Nrf2/HO-1 in determining sensitivity [20,31].

Network-based interactome analysis identified PIK3R1, SRC, and ESR1 as highly connected nodes within cancer-associated signaling networks. These hubs converge on the PI3K/Akt pathway, a critical regulator of NSCLC cell proliferation, survival, and migration [32]. To contextualize these findings, we employed three NSCLC cell lines well-validated in in vitro models for lung cancer biology and drug response. Molecular docking simulations revealed that isalpinin, a methylated flavonol, binds directly to the ATP-binding pocket of PIK3R1 (p85α subunit of PI3K), suggesting its potential as a PI3K/Akt pathway inhibitor. Experimental validation in H460 cells confirmed that isalpinin treatment reduces AKT (Ser473) and GSK3β (Ser9) phosphorylation, consistent with pathway inhibition. The structural basis for this interaction lies in the isalpinin planar flavonol scaffold, which facilitates π–π stacking with aromatic residues (e.g., Phe439, Tyr231) and hydrogen bonding via hydroxyl/methoxy groups (e.g., Glu236, Thr292). Density functional theory calculations further support the coplanarity of rings A, B, and C in methylated flavonols like isalpinin, enhancing electron delocalization and binding affinity [33,34]. To broaden the mechanistic insights, we integrated NSCLC-specific transcriptomic datasets (TCGA LUAD/LUSC cohorts) and pathway enrichment analysis (GO/KEGG). These analyses highlighted PI3K/Akt signaling as a key dysregulated pathway in NSCLC, aligning with isalpinin predicted targets from STRING DB and DrugBank. Flavonols have been reported to interact with PI3K/Akt components*, reinforcing the relevance of the mechanism of isalpinin in NSCLC pathophysiology [35].

Nuclear morphology assays using Hoechst 33342 staining revealed that isalpinin induced apoptosis in all three cell lines in a dose- and time-dependent manner, with the highest levels observed in H460 cells, followed by H23 and A549 cells. Specifically, H460 cells exhibited apoptosis rates approaching 80% after 48 h at 40 µM of isalpinin, indicating potent pro-apoptotic activity. A Western blot analysis further confirmed the downregulation of Bcl-2, an anti-apoptotic member of the Bcl-2 family, in both H23 and H460 cells. The suppression of Bcl-2 aligns with the activation of the intrinsic (mitochondrial) apoptotic pathway, as Bcl-2 plays a crucial role in maintaining the mitochondrial membrane integrity and preventing cytochrome c release. Its downregulation likely facilitates the activation of the apoptotic cascade via caspase-9 and -3. Supporting this, isorhamnetin was shown to inhibit the PI3K/Akt/GSK3β axis in hepatocellular carcinoma. It induces apoptosis via mitochondrial pathways by increasing pro-apoptotic proteins (Bax and cleaved caspases) and suppressing anti-apoptotic Bcl-2 without activating ROS-mediated mechanisms. The apoptotic effects were entirely PI3K/Akt dependent, as shown by the consistent downregulation of pathway activity and effective suppression of tumor growth in vivo [33]. These results contrast with those of kaempferide, which exerts anticancer effects via the ROS-dependent activation of JNK and the suppression of PI3K/Akt and quercetin, which disrupt mitochondrial integrity via ROS accumulation and Sestrin2-AMPK activation [20]. While Hoechst staining effectively demonstrated characteristic apoptotic morphology, including chromatin condensation and nuclear fragmentation, we acknowledge that a direct measurement of cleaved caspase-3 would provide additional mechanistic insight into apoptotic pathway activation [36]. Hoechst 33342 staining is a well-established and reliable method for detecting apoptosis based on nuclear morphological changes, which represent fundamental hallmarks of early apoptosis [37,38]. In our study, significant Bcl-2 downregulation (Figure 7G,H), together with the characteristic apoptotic morphological changes observed by Hoechst staining (Figure 7A,C,E), provide multiple independent lines of evidence supporting apoptosis induced by isalpinin. The dose-dependent relationship between isalpinin treatment and both morphological changes and Bcl-2 expression further supports this conclusion. The observed nuclear condensation and fragmentation are consistent with classical apoptotic cell death, while concurrent Bcl-2 downregulation indicates activation of the mitochondrial-mediated apoptotic pathway.

The temporal progression of apoptotic events explains the differences observed between Hoechst fluorescence at 20 μM (reflecting early apoptosis) and Bcl-2 downregulation at 40 μM (reflecting late apoptosis). As highlighted by Guo et al. (2021) [29], morphological changes detected by Hoechst staining occur earlier than biochemical markers such as Bcl-2 degradation. Additionally, methodological differences in cell density between assays may influence drug sensitivity: lower-density cultures used for Hoechst staining are more susceptible to apoptosis initiation, whereas higher-density cultures required for Western blot analysis may need stronger pro-apoptotic stimuli to overcome survival signals. Despite these variations, both experiments consistently demonstrate a dose-dependent induction of apoptosis by isalpinin, with an alignment of morphological and biochemical hallmarks at higher concentrations. This body of evidence underscores that PIK3R1 is a key modulator and pharmacological target of flavonol-based compounds. Although isorhamnetin and isalpinin inhibit the PI3K/Akt pathway through direct or structural binding, their methylation profiles enhance both metabolic stability and binding selectivity [39]. This provides a compelling rationale for the development of methylated flavonols as non-toxic, multi-target anticancer agents.

Based on prior evidence implicating the Akt pathway in the mechanism of isalpinin, its direct impact on signaling was assessed in responsive NSCLC lines. The selection of H23 and H460 cells for this analysis was guided by their differential sensitivities and underlying genetic profiles. These cell lines demonstrated a high sensitivity to isalpinin, with an IC_50_ approximately 2-fold lower than that of A549 at 48 h. This lower sensitivity of A549 cells is consistent with the presence of a KRAS mutation, which is associated with the dominance of MAPK signaling and a reduced dependency on the PI3K/Akt pathway [40]. Conversely, the high basal p-Akt levels in H460 cells and the loss of STK11/LKB1 in H23 cells created a greater reliance on PI3K/mTOR pathway signaling [41,42], thereby enhancing their sensitivity to inhibition by isalpinin. In this study, isalpinin demonstrated in vitro anticancer activity by inhibiting the PI3K/Akt pathway through ATP-binding site interaction similar to alpelisib and by generating ROS, reflecting established NSCLC therapeutics [43,44]. A concentration-dependent suppression of Akt (Ser473) and GSK3β (Ser9) phosphorylation was observed, targeting a pathway frequently dysregulated in NSCLC [45]. Additionally, isalpinin generates reactive oxygen species ROS through a mechanism analogous to cisplatin [45,46], achieving potent cytotoxicity (IC_50_ 44 to 82 μM) while demonstrating superior cancer cell selectivity over normal fibroblasts (IC_50_ > 100 μM vs. cisplatin 14.65 μM) in cell-based assays. This preferential activity in H23/H460 lines correlates with *PIK3CA* alterations prevalent in 33.1% of lung squamous cell carcinomas [42] which sensitize tumors to PI3K/Akt pathway modulation [47]. While these in vitro results highlight the potential of isalpinin as an anticancer candidate, further in vivo validation remains essential to confirm therapeutic efficacy. These preliminary findings suggest that isalpinin is a promising candidate for drug discovery development and warrants continued investigation as a potential therapeutic agent to address the current limitations in NSCLC treatment.

Combination therapeutic strategies are paramount in contemporary oncology, aimed at overcoming drug resistance, mitigating adverse effects, and improving patient outcomes. Flavonoids exhibit considerable promise as both monotherapeutic agents and as adjuvants that enhance the efficacy of conventional chemotherapy or immunotherapy. For instance, quercetin and kaempferol augment the cytotoxic effects of cisplatin by impeding DNA repair pathways (e.g., base excision repair) and inhibiting drug efflux pumps (e.g., P-glycoprotein), thereby increasing intracellular platinum accumulation [48,49]. The synergistic potential of flavonols with immune checkpoint inhibitors is currently being investigated in clinical trials, as exemplified by the ongoing Phase 2 study (ClinicalTrials.gov identifier: NCT05724329) evaluating neoadjuvant tislelizumab combined with dasatinib and quercetin in resectable head and neck squamous cell carcinoma (COIS-01). The distinct mechanisms of action of isalpinin, characterized by PI3K/Akt pathway inhibition and reactive oxygen species-driven apoptosis, position it as a promising combinatorial agent, potentially facilitating reduced cisplatin dosages, thereby diminishing nephrotoxicity while preserving efficacy, and could act in concert with chemotherapeutic drugs within the tumor microenvironment. Integrating flavonoid-driven strategies is crucial for addressing critical challenges in precision oncology, particularly in tumors with PI3K/Akt dysregulation or immunosuppressive tumor microenvironments. Future research needs to be conducted to validate the synergy in relevant in vitro models (e.g., H23 and H460: NSCLC subtypes) and in vivo-resistant tumor models, and to optimize doses for efficacy and safety in flavonoid-chemotherapy and flavonoid-immune checkpoint inhibitor pairings.

This study is the first to demonstrate that isalpinin from *P. dianthum* acts as a multi-target subtype-selective anticancer agent against non-small cell lung cancer (NSCLC). This shows that isalpinin inhibits cell proliferation, migration, and anchorage-independent growth; induces ROS-mediated apoptosis; and suppresses the PI3K/Akt/GSK3β signaling pathway, resulting in cell death via apoptosis. These findings provide novel mechanistic insights and suggest the potential of isalpinin for the preclinical development of lung cancer therapy.

## 4. Materials and Methods

### 4.1. Materials

RPMI-1640, DMEM, L-glutamine, penicillin, streptomycin, fetal bovine serum (FBS), 0.25% trypsin-EDTA, and phosphate-buffered saline (PBS) were purchased from GIBCO (Gaithersburg, MA, USA). A quantity of 3-(4,5-dimethylthiazol-2-yl)-2,5-diphenyltetrazolium bromide (MTT) was purchased from Invitrogen (Carlsbad, CA, USA). Dimethyl sulfoxide (DMSO), Hoechst 33342, 2′-7′- dichlorodihydrofluorescein diacetate (DCFH_2_-DA) were obtained from Sigma (St. Louis, MO, USA). Agar nobles were purchased from BD Biosciences (Franklin Lakes, NJ, USA). Primary antibodies were used as follows: Rabbit anti-phosphorylated Akt (Ser473; CST, Beverly, MA, USA); rabbit anti-Akt (CST, Beverly, MA, USA); rabbit anti-phosphorylated GSK-3β (Ser9; CST, Beverly, MA, USA); rabbit anti-GSK-3β (CST, Beverly, MA, USA); rabbit anti-GAPDH (CST, Beverly, MA, USA) and rabbit anti-Bcl-2 (CST, Beverly, MA, USA). The secondary antibody used in this study was anti-rabbit IgG HRP-linked (CST, Beverly, MA, USA).

### 4.2. Methods

#### 4.2.1. Isalpinin Preparation

Isalpinin (Figure 1, Supplementary S1) was isolated from the roots and leaves of *P. dianthum* as previously described [13]. For isalpinin preparation, it was dissolved in DMSO as a stock solution, which was further diluted with cell culture medium to the desired working concentrations, and the control samples were incubated with 0.1% DMSO in the culture medium. The final concentration of DMSO was less than 0.1%, indicating no toxicity. Control cells exposed to equal concentrations of DMSO were used for comparison with the effect of the isalpinin-treated group.

#### 4.2.2. Cell Culture

Human non-small cell lung cancer H460 (HTB-177), H23 (CRL-5800), A549 (CCL-185), as well as the normal mouse embryonic fibroblast cell line NIH/3T3 (CRL-1658) were obtained from American Type Culture Collection (ATCC) (Manassas, VA, USA). H460 and H23 cells were cultured in RPMI-1640 medium and A549 cells were cultured in DMEM (Gibco, Gaithersburg, MA, USA). All cell cultures were supplemented with 10% fetal bovine serum (FBS), 2 mM L-glutamine, and 100 units/mL penicillin/streptomycin and incubated at 37 °C in a humidified atmosphere of 5% CO_2_ and 95% humidity stream up to 70–80% confluence before use in the experiments.

#### 4.2.3. Cytotoxicity and Cell Proliferation Assays

The cytotoxic effects of isalpinin were assessed using the MTT colorimetric assay, following the method previously described by [50]. Briefly, cells were seeded in 96-well plates at a density of 1 × 10^4^ cells per well and allowed to adhere overnight at 37 °C in a humidified incubator with 5% CO_2_. The following day, the cells were treated with increasing concentrations of isalpinin (0–200 μM) or cisplatin (0–250 μM), as a positive control for 24 and 48 h. After the treatment period, the cells were washed with phosphate-buffered saline (PBS), and 100 μL of MTT solution (0.5 mg/mL) was added to each well. The plates were incubated for an additional 3 h to allow formazan crystal formation. Subsequently, 200 μL of DMSO was added to each well to solubilize the crystals, and absorbance was measured at 570 nm using a microplate reader (Perkin Elmer VICTOR3/Wallac1420). Cell viability was calculated as a percentage of the untreated control, and the IC_50_ values (concentration required to inhibit 50% of cell viability) were determined using nonlinear regression analysis in GraphPad Prism version 8 (GraphPad Software, CA, USA).

To assess cell proliferation, a low seeding density of 2 × 10^3^ cells per well was used in 96-well plates. Cells were allowed to adhere overnight and were subsequently treated with a non-cytotoxic concentration of isalpinin for 48 h, encompassing approximately two cell doubling times. Cell growth was measured using the MTT assay protocol. The results were expressed as the percentage of cell proliferation in the treated wells relative to that in the control wells.

#### 4.2.4. Cell Migration Assays

Cell migratory capacity was evaluated using a wound-healing assay, as previously described with modifications [38,51]. The assay was employed to assess the inhibitory effect of isalpinin on the migration of non-small cell lung cancer cell lines H23, H460, and A549. Briefly, cells were seeded into 24-well plates at a density of 2 × 10^5^ cells per well and allowed to adhere overnight in a complete culture medium. Upon reaching confluence, a uniform linear scratch was introduced across the monolayer using a sterile 200 μL pipette tip to simulate a wound. The detached cells and debris were carefully removed by washing with PBS buffer 500 μL. Subsequently, the cells were treated with varying concentrations of isalpinin (0–40 μM) and incubated for several hours. Wound closure was monitored using phase-contrast microscopy with a Nikon Ts2 (Tokyo, Japan) digital microscope. Images were captured 0, 24, and 48 h post-scratch. The wound area was quantified using ImageJ software version 1.53 (National Institutes of Health, Bethesda, USA), and the relative wound area was recalculated. The rate of cell migration was determined by calculating the value from the regression analysis of wound closure area the 48 h period. All experiments were conducted in triplicate to ensure reproducibility.

#### 4.2.5. Soft Agar Colony Formation Assay

The ability of isalpinin to inhibit anchorage-independent growth and survival of non-small cell lung cancer cell lines (A549, H23, and H460) was evaluated using a soft agar colony formation assay, as previously, described with modifications [52]. A two-layer soft agar system was established in 24-ultra-low-attachmenthment plates (Corning, NY, USA). The bottom layer consisted of 250 μL of 1.2% Noble agar (Sigma-Aldrich, Louis, MO, USA) prepared in DMEM or RPMI-1640 medium supplemented with 5% FBS. This layer was allowed to dry at 37 °C for 2 h. The top layer was composed of 0.6% agar containing 1.5 × 10^3^ cells per well (A549, H23, or H460) in the same medium, supplemented with either a non-cytotoxic concentration of isalpinin or 0.1% (*v*/*v*) DMSO as a vehicle control. Following solidification of the top agar layer, 250 μL of complete medium was added to each well to maintain hydration and nutrient availability. The cultures were incubated at 37 °C in a humidified incubator with 5% CO_2_ for 14 days. The culture medium was carefully replenished every 3 days. At the end of the incubation period, the colonies were visualized using a light microscope equipped with a digital camera. Images were captured and analyzed to quantify both colony number and size, which reflect the extent of anchorage-independent cell proliferation. A quantitative analysis was performed using Axiovision 4 software (Carl Zeiss MicroImaging GmbH, Jena, Germany) and I (Institute of Health, Bethesda, MD, USA).

#### 4.2.6. Determination of Reactive Oxygen Species (ROS)

Intracellular reactive oxygen species (ROS) levels were quantified using the DCFH_2_-DA assay [37]. Briefly, cells (7.5 × 10^4^ cells/well) were seeded as a monolayer in 24-well plates and incubated overnight. After washing with PBS, the cells were incubated with 100 μM of DCFH_2_-DA solution for 30 min. at 37 °C. Subsequently, the cells were treated with isalpinin (0–40 μM) or 600 μM hydrogen peroxide (H_2_O_2_) as a positive control for 1 h. After treatment, the cells were lysed with 1% Triton X-100 and the fluorescence intensity of oxidized DCF was measured at 485 nm excitation and 535 nm emission using a microplate reader (Perkin Elmer VICTOR3/Wallac1420).

#### 4.2.7. Cell Apoptosis

Apoptotic cell death was evaluated using Hoechst 33342, a fluorescent nuclear staining dye [36]. A549, H23, and H460 cells were seeded at a density of 8 × 10^3^ cells/well in 96-well plates and allowed to adhere overnight. The cells were treated with isalpinin for 48 h. Following treatment, cells were incubated with Hoechst 33342 (10 μg/mL) for 30 min in the dark at room temperature. Nuclear morphological changes characteristic of apoptosis, including chromatin condensation and DNA fragmentation, were visualized using a fluorescence microscope (BX-FLA; Olympus, Tokyo, Japan) at 20× magnification with excitation/emission wavelengths of 350/461 nm. Apoptotic cells were quantified and expressed as a percentage of the total cell population. All experiments were conducted in triplicate to ensure reproducibility.

#### 4.2.8. Western Blot Analysis

A total of 8 × 10^5^ cells were seeded into 60-mm culture dishes and incubated overnight to allow attachment. The cells were treated with various concentrations of isalpinin for 24 h. Following treatment, cells were lysed on ice for 30 min using LIPA buffer supplemented with a protease inhibitor cocktail (Corning, NY, USA). Lysates were clarified by centrifugation at 12,000× *g* for 15 min at 4 °C and the resulting supernatants were collected. The Total protein concentration was determined using a BSA protein assay kit (Thermo Fisher Scientific, Waltham, MA, USA). The protein samples were denatured by boiling in 2X sample buffer at 95 °C for 5 min. Equal amounts of protein (40 μg per lane) were separated using SDS-polyacrylamide gel electrophoresis (SDS-PAGE) and subsequently transferred onto polyvinylidene fluoride (PVDF) membranes. Membranes were blocked in 5% skim milk prepared in Tris-buffered saline with 0.1% Tween-20 (TBS-T) for 1 h at room temperature, followed by overnight incubation at 4 °C with rabbit primary antibodies. After washing, the membranes were incubated with appropriate HRP-conjugated secondary antibodies at room temperature for 2 h. Glyceraldehyde-3-phosphate dehydrogenase (GAPDH) was used as the loading control. Protein bands were visualized using enhanced chemiluminescence (ECL) with Immobilon Western Chemiluminescent HRP Substrate (Millipore, MA, USA) and quantified using ImageJ software (NIH, Bethesda, MD, USA).

#### 4.2.9. Gene Ontology (GO) and Kyoto Encyclopedia of Genes and Genomes (KEGG) Analysis

Transcriptomic data from NSCLC cell lines were analyzed alongside public NSCLC datasets (TCGA) to identify dysregulated pathways. GO [53] and KEGG [54] analyses were performed using the STRING 11.5 database [55], with relationships between gene count and adjusted *p*-value visualized via scatterplots. R software version 4.2.3 with ggplot2 [56] was used to generate bubble scatterplots of enriched GO terms and KEGG pathways [57]. Potential targets of isalpinin were predicted using molecular docking against proteins in the PI3K/Akt pathway, validated against structural data from PubChem and activity profiles in ChEMBL. Functional annotation and statistical analyses employed standard bioinformatics tools, with *p*-values < 0.05 as the significance threshold. These integrated analyses provided mechanistic insights into the multitarget effects of isalpinin, highlighting its regulation of key biological processes (e.g., apoptosis, proliferation) and signaling pathways (e.g., PI3K/Akt) relevant to NSCLC.

#### 4.2.10. Construction of the Protein–Protein Interaction (PPI) Network and Computational Modeling of AKT with Molecular Docking

To investigate the protein interaction landscape, a protein–protein interaction (PPI) network was constructed using the STRING database (version 11.0; [55] https://string-db.org/) [58]. Gene targets of interest were input under the Homo sapiens organism model and a high-confidence interaction score threshold of 0.950 was applied. In the resulting network, nodes represented proteins and edges denoted by protein–protein associations [59]. To identify core target proteins, topological parameters, including degree centrality, betweenness centrality, closeness centrality, and clustering coefficient, were calculated and analyzed using Cytoscape software.

For molecular docking analysis, the X-ray crystallographic structure of activated Akt was retrieved from the Protein Data Bank (PDB ID: 1O6L). The chemical structure of isalpinin was generated using ChemDraw Ultra 17.0 (PerkinElmer, Waltham, MA, USA) and the canonical SMILES format was obtained from the ChEBI database [60]. The potential binding interactions between isalpinin and AKT were evaluated using molecular docking simulations. To determine the docking site, CurPocket, a curvature-based cavity detection algorithm, was used to identify the potential binding pockets on the AKT protein surface. Molecular docking was subsequently performed using AutoDock Vina (version 1.1.2) integrated into the CB-Dock2 platform, which enables blind docking simulations [61]. The binding poses with the most negative Vina score, which is indicative of the highest binding affinity, was selected for further analysis. Detailed binding parameters, including the docking center, grid size, interacting residues, and molecular contacts, were examined. The resulting protein–ligand interactions and docking conformations were visualized using BIOVIA Discovery Studio Visualizer (BIOVIA, San Diego, CA, USA), providing structural insight into the binding mechanism of isalpinin with AKT.

#### 4.2.11. Statistical Analysis

Data are presented as mean ± standard error of the mean (SEM) from at least four independent experiments, and all data were analyzed using Prism 8 (GraphPad Software, Inc., San Diego, CA, USA). One-way analysis of variance (ANOVA) with Tukey’s Multiple Comparison Test was used to determine statistical significance between the control and treatment groups, with *p*-values < 0.05.

## 5. Conclusions

In summary, this study demonstrates that isalpinin, a flavonol derived from *P. dianthum*, exhibits potent anticancer activity against non-small cell lung cancer (NSCLC) cell lines. At concentrations of 40 μM, isalpinin significantly inhibited cell viability, proliferation, and migration, while promoting apoptosis particularly in H23 and H460 cells. Mechanistically, isalpinin induced apoptosis via ROS generation, Bcl-2 downregulation, and the suppression of the Akt/GSK3β signaling pathway. These findings align with growing evidence supporting the role of natural flavonoids in modulating key oncogenic pathways in lung cancer. The marked sensitivity of H23 and H460 cells underscores the potential of isalpinin as a subtype-specific therapeutic candidate for NSCLC. Further in vivo validation and combination studies with standard therapies are warranted to advance the clinical development of isalpinin as a novel anticancer agent.

## Figures and Tables

**Table 1:** IC_50_ values of isalpinin and cisplatin against NSCLC cell lines and mouse fibroblast embryonic cell lines following 24 h and 48 h exposure.

NSCLC Cell Lines/Normal Mouse Fibroblast Embryonic Cell Lines	^a^ IC_50_ of Isalpinin (μM ± SEM)		^a^ IC_50_ of Cisplatin (μM ± SEM)	
	24 h	48 h	24 h	48 h
A549	105.2 ± 14.46	81.88 ± 23.36	48.03 ± 4.71 *	19.17 ± 3.34 *
H23	100.3 ± 24.39	44.34 ± 16.34 *	86.34 ± 8.91	33.39 ± 2.95 *
H460	103.1 ± 29.42	44.46 ± 13.40 *	66.55 ± 7.59	41.43 ± 8.58 *
NIH/3T3	>100	>100	36.25 ± 5.64	14.65 ± 2.58

^a^ Concentration that inhibits cell viability by 50%. Data are expressed as the mean ± standard error of the mean (SEM) (*n* = 3). * *p* < 0.05 vs. untreated control cells.

## Data Availability

The datasets generated during and/or analyzed during the current study are available by request; please contact the corresponding authors.

## Supplementary Materials

The following supporting information can be downloaded at: https://www.mdpi.com/article/10.3390/molecules30132762/s1, Figure S1: HR-ESI mass spectrum of compound PD-1; Figure S2: ^1^H NMR spectrum of compound PD-1 (300 MHz, in CDCl3); Figure S3: ^13^C NMR spectrum of compound PD-1 (75 MHz, in CDCl3); Figure S4: Intercept targets between compound’s targets and non-small cell lung cancer’s targets by Venny; Figure S5: Gene Ontology (GO) enrichment analysis based on biological process, molecular function, and cellular component. The data are ranged from the highest significant on the top to those of lower at the bottom; Figure S6: KEGG pathway analysis. The data are ranged from the highest significant on the top to those of lower at the bottom; Figure S7: The top targets in the PPI network as ranked using the cytoHubba plug in network analyzer. The higher degree value is represented by colors ranging from purple to blue; Figure S8: Isalpinin directly binds to activated Akt. (a) Molecular docking analysis of the binding Pocket C1 of activated Akt and Isalpinin was performed by CB-Dock2. (b) The binding pose, contact residues, and molecular interactions were visualized using BIOVIA discovery studio; Table S1: NMR spectral data of compound PD-1 and isalpinin (in CDCl3); Table S2: Target prediction of Isalpinin by Swisstargetprediction Tool; Table S3: Target prediction of Dihydroxy methoxystillbene by Similarity ensemble approach (SEA) Tool; Table S4: Pharmacokinetic prediction by pkCSM Tool; Table S5: List of intercept targets between compound and non-small cell lung cancer cell targets by Venny; Table S6: Important nodes in network analyzer; Table S7: CurPocket-based information on the five largest binding pockets of pAkt protein; Table S8: Binding pockets with the greatest negative Vina scores after blind docking with Isalpinin.
