# Supplementary material for: Antitumor Activity of Isalpinin from Paphiopedilum dianthum on Non-Small Cell Lung Cancer Cell Lines
Source: Molecules. 2025 Jun 27;30(13):2762. doi: 10.3390/molecules30132762 (PMC12251045; doi:10.3390/molecules30132762)
Supplement: Supplementary file 1 [file molecules-30-02762-s001.zip › molecules-3667673-supplementary.pdf]

Supplementary 1 (S1)

Identification of compound PD-1 (isalpinin) and NMR spectral data of compound PD-1 and isalpinin (in CDCl3)

1. Identification of compound PD-1 (isalpinin)

Compound PD-1, obtained as yellow needles (48 mg, 0.012% yield from the dried roots and 7 mg, 0.0012% yield from the dried leaves of *Paphiopedilum dianthum*), appeared as a yellow spot on TLC upon spraying with 10% sulfuric acid and heating, suggesting this compound could be a flavonoid. Its molecular formula of $C_{16}H_{12}O_5$ was analyzed from the $[M + Na]^+$ ion peak at 307.0579 *m/z* (calcd. 307.0582) in its high resolution ESI-TOF mass spectrum (**Figure S1**).

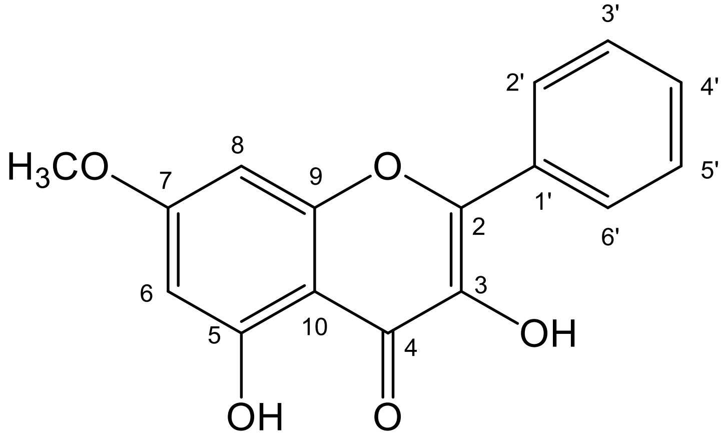

Isalpinin

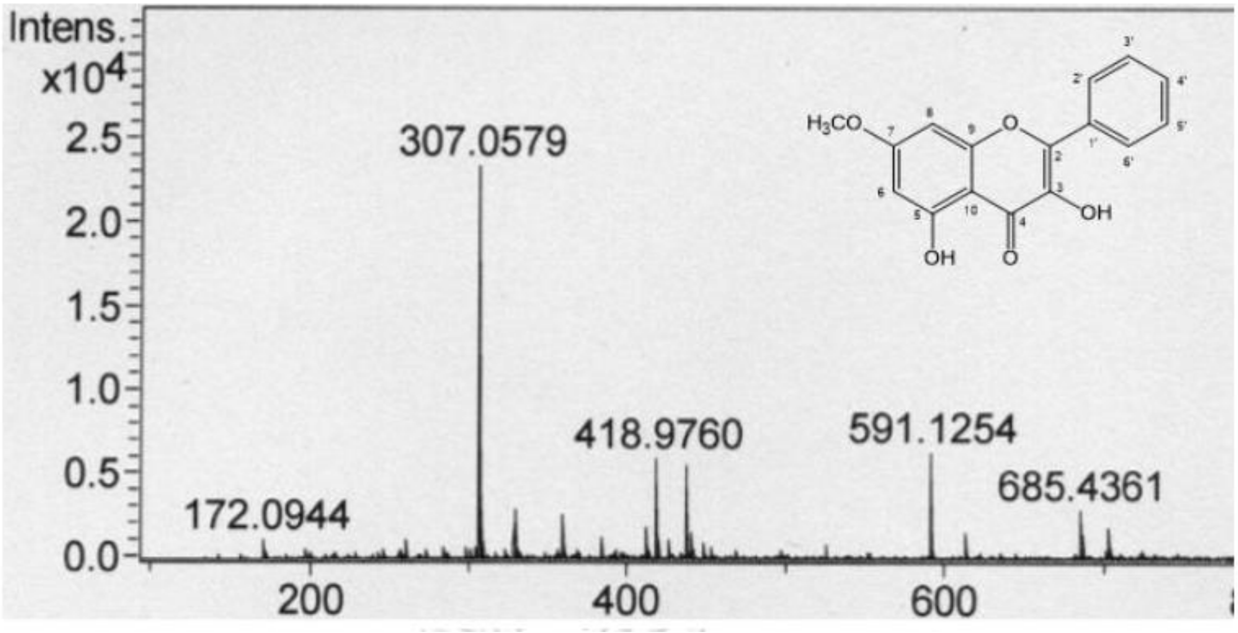

Figure S1. HR-ESI mass spectrum of compound PD-1

The ^{1}H NMR spectrum of compound PD-1 (**Figure S2** and **Table S1**) showed a methoxy signal at δ 3.90 ppm (3H, *s*, 7-OCH3) and two broad hydroxy singlets at δ 6.66 (1H, *br s*, 3-OH) and δ 11.67 ppm (1H, *s*, 5-OH). The downfield shift of the latter signal indicated the position of one hydroxy group at position 5 of flavonoid nucleus. A pair of doublets at δ 6.39 (1H, *d*, *J* = 2.1 Hz) and 6.52 ppm (1H, *d*, *J* = 2.1 Hz) represented the *meta*-coupled aromatic protons of positions 6 and 8, respectively, on ring A of a flavonoid. Three aromatic proton resonances, integrated for five protons, at δ 7.51 (2H, *m*, H-3'/H-5'), 7.53 (1H, *m*, H-4') and 8.20 ppm (2H, *dd*, *J* = 8.4, 1.5 Hz, H- 2'/H-6') were characteristic of the unsubstituted ring B of flavonoids. Absence of other proton signals, especially those of positions 2 and 3 was suggestive of a flavonol structure. Therefore, compound PD-1 should be a flavonol with one hydroxy substitution at C-5 and one methoxy substitution at C-7.

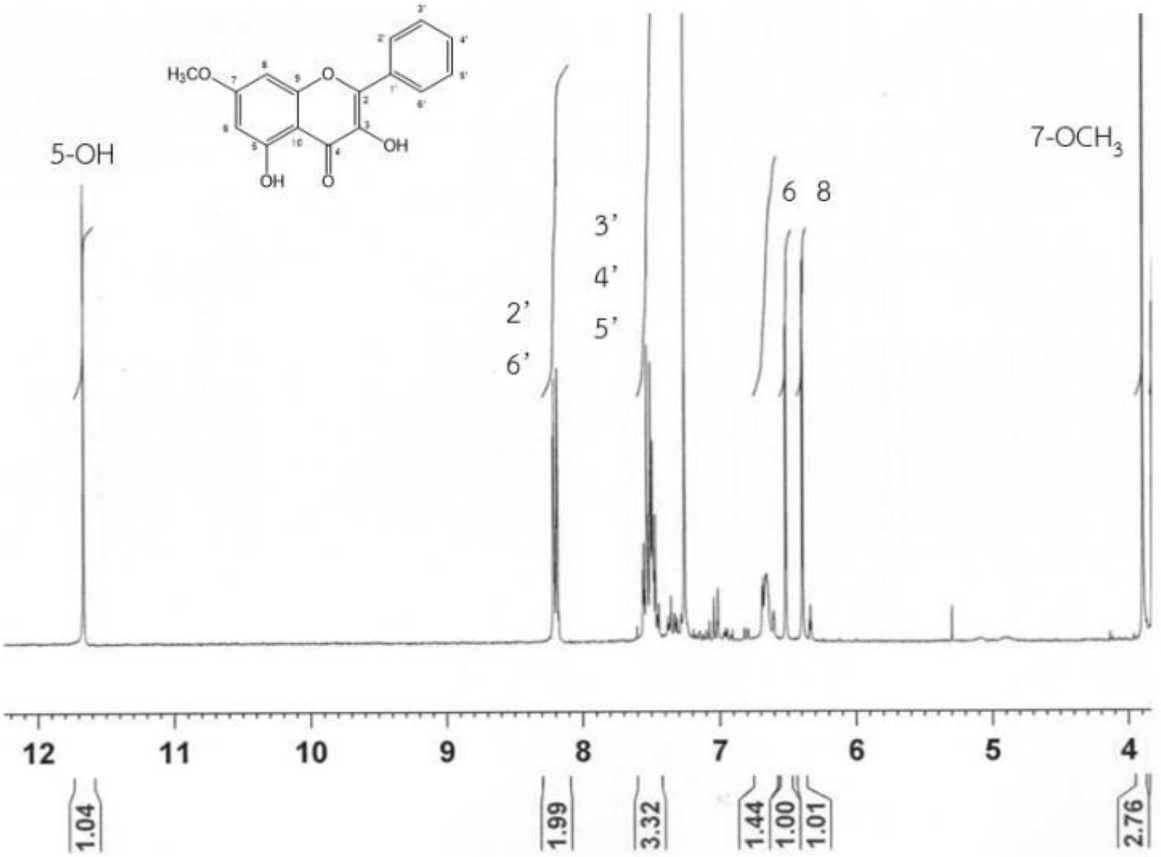

Figure S2. ^{1}H NMR spectrum of compound PD-1 (300 MHz, in $CDCl_3$)

The ^{13}C spectrum of compound PD-1 (**Figure S3** and **Table S1**) displayed sixteen carbon resonances including those of a methoxy carbon at δ 55.9 ppm (7- OCH3), a carbonyl carbon at δ 175.5 ppm (C-4) and an unsubstituted ring B at δ 127.6 (C-2'/C-6'), 128.6 (C-3'/C-5'), 130.3 (C-4') and 130.7 ppm (C-1'). Two quaternary carbon signals at δ 145.1 (C-2), 136.6 ppm (C-3) and the carbonyl C-4 peak at δ 175.5 ppm were supportive of its flavonol nature (4). Comparison of these NMR data with previously reported values (210) led to the identification of compound PD-1 as isalpinin (5-hydroxy-7-methoxyflavonol). This flavonoid has been found in propolis (royal jelly) from several sources (210-212). In addition, it could be extracted from plants such as *Polygonum hydropiper* (Family Polygonaceae) (213), *Chromolaena leivensis* and *C. tacotana* (family Asteraceae) (214, 215).

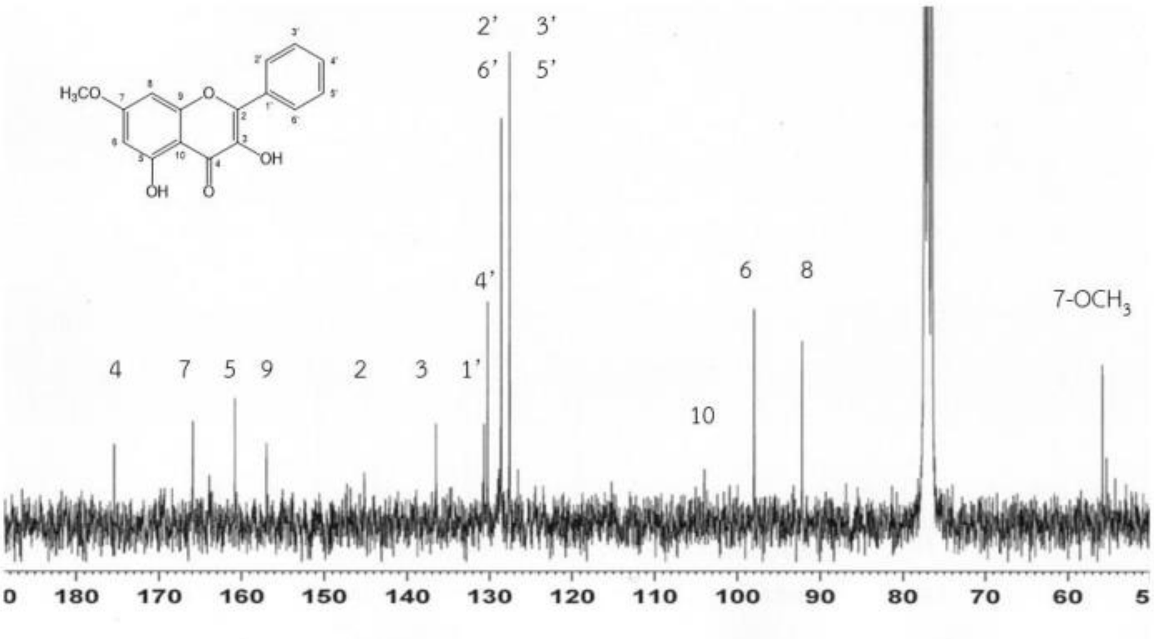

Figure S3. ^{13}C NMR spectrum of compound PD-1 (75 MHz, in $CDCl_3$)

2. NMR spectral data of compound PD-1 and isalpinin (in CDCl3)

Table S1. NMR spectral data of compound PD-1 and isalpinin (in $CDCl_3$)

Position	Compound PD-1		Isalpinin[210]	
	δ_H (mult., *J* in Hz)	δ_C	δ_H (mult., *J* in Hz)	δ_C
2	-	145.1	-	145.0
3	-	136.6	-	136.0
4	-	175.5	-	176.1
5	-	160.9	-	160.8
6	6.39 (*d*, 2.1)	98.0	6.35 (*d*, 2.0)	97.8
7	-	166.0	-	164.3
8	6.52 (*d*, 2.1)	92.3	6.45 (*d*, 2.0)	92.0
9	-	157.0	-	156.5
10	-	104.2	-	103.2
1'	-	130.7	-	130.9
2'	8.20 (*dd*, 8.4, 1.5)	127.6	8.15 (*dd*, 8.0, 1.5)	126.9
3'	7.51 (*m*)	128.6	7.50 (*m*)	128.0
4'	7.53 (*m*)	130.3	7.52 (*m*)	130.0
5'	7.51 (*m*)	128.6	7.50 (*m*)	128.0
6'	8.20 (*dd*, 8.4, 1.5)	127.6	8.15 (*dd*, 8.0, 1.5)	126.9
7-OCH_3	3.90 (*s*)	55.9	3.75 (*s*)	55.2
3-OH	6.66 (*br s*)	-	-	-
5-OH	11.67 (*s*)	-	11.61 (*s*)	-

จุฬาลงกรณ์มหาวิทยาลัย
CHULALONGKORN UNIVERSITY

Table S2. Target prediction of Isalpinin by Swisstargetprediction Tool

Tyrosine-protein kinase receptor FLT3	FLT3	Carbonic anhydrase II	CA2
P-glycoprotein 1	ABCB1	Epidermal growth factor receptor erbB1	EGFR
Nitric oxide synthase, inducible	NOS2	Interleukin-8 receptor A	CXCR1
Xanthine dehydrogenase	XDH	Death-associated protein kinase 1	DAPK1
PI3-kinase p110-gamma subunit	PIK3CG	DNA-3-methyladenine glycosylase	MPG
ATP-binding cassette sub-family G member 2	ABCG2	Induced myeloid leukemia cell differentiation protein Mcl-1	MCL1
Cyclooxygenase-2 (by homology)	PTGS2	Lysine-specific demethylase 4D-like	KDM4E
Cytochrome P450 1B1	CYP1B1	Cyclin-dependent kinase 1	CDK1
Solute carrier family 22 member 12	SLC22A12	Arachidonate 12-lipoxygenase	ALOX12
Estradiol 17-beta-dehydrogenase 2	HSD17B2	Plasminogen	PLG
Estradiol 17-beta-dehydrogenase 1	HSD17B1	Hepatocyte growth factor receptor	MET
Estrogen receptor beta	ESR2	Microtubule-associated protein tau	MAPT
Carbonic anhydrase VII	CA7	DNA topoisomerase II alpha	TOP2A
Carbonic anhydrase XII	CA12	Insulin receptor	INSR
Carbonic anhydrase IV	CA4	Myosin light chain kinase	MYLK
Arachidonate 15-lipoxygenase	ALOX15	Myeloperoxidase	MPO
Tyrosinase	TYR	PI3-kinase p85-alpha subunit	PIK3R1
Aryl hydrocarbon receptor	AHR	Liver glycogen phosphorylase	PYGL
Estrogen-related receptor alpha	ESRRA	Focal adhesion kinase 1	PTK2
Butyrylcholinesterase	BCHE	Matrix metalloproteinase 13	MMP13
Acetylcholinesterase	ACHE	Matrix metalloproteinase 3	MMP3
Adenosine A1	ADORA1	Carbonic anhydrase III	CA3
Adenosine A2a	ADORA2A	Carbonic anhydrase XIV	CA14
Adenosine A3 receptor	ADORA3	Serine/threonine-protein kinase NEK2	NEK2
Aldose reductase	AKR1B1	Carbonic anhydrase XIII	CA13

Table S3. Target prediction of Dihydroxy methoxystillbene by Similarity ensemble approach (SEA) Tool

Cyclic AMP-responsive element-binding protein 1	CREB1	Taste receptor type 2 member 31	TAS2R31
ELAV-like protein 3	ELAVL3	Xanthine dehydrogenase/oxidase	XDH
Cytochrome P450 1B1	CYP1B1	Protein disulfide-isomerase	P4HB
Tyrosine-protein phosphatase non-receptor type 9	PTPN9	Tyrosine-protein phosphatase non-receptor type 6	PTPN6
Receptor-type tyrosine-protein phosphatase S	PTPRS	Low molecular weight phosphotyrosine protein phosphatase	ACP1
Cystathionine beta-synthase	CBS	ATP-dependent translocase ABCB1	ABCB1
Broad substrate specificity ATP-binding cassette transporter ABCG2	ABCG2	DNA-3-methyladenine glycosylase	MPG
Low-density lipoprotein receptor-related protein 6	LRP6	ELAV-like protein 1	ELAVL1

Table S4. Pharmacokinetic prediction by pkCSM Tool

Property	Model Name	Predicted Value	Unit
Absorption	Water solubility	-3.469	Numeric (log mol/L)
	Caco2 permeability	0.955	Numeric (log Papp in 10^{-6} cm/s)
	Intestinal absorption	94.905	Numeric (% Absorbed)
	Skin Permeability	-2.74	Numeric (log Kp)
	P-glycoprotein substrate	Yes	Categorical (Yes/No)
	P-glycoprotein I inhibitor	No	Categorical (Yes/No)
	P-glycoprotein II inhibitor	No	Categorical (Yes/No)
Distribution	VDss	-0.186	Numeric (log L/kg)
	Fraction unbound	0.058	Numeric (Fu)
	BBB permeability	-0.093	Numeric (log BB)
	CNS permeability	-2.174	Numeric (log PS)
Metabolism	CYP2D6 substrate	No	Categorical (Yes/No)
	CYP3A4 substrate	Yes	Categorical (Yes/No)
	CYP1A2 inhibitior	Yes	Categorical (Yes/No)
	CYP2C19 inhibitior	Yes	Categorical (Yes/No)
	CYP2C9 inhibitior	Yes	Categorical (Yes/No)
	CYP2D6 inhibitior	No	Categorical (Yes/No)
	CYP3A4 inhibitior	Yes	Categorical (Yes/No)
Excretion	Total Clearance	0.323	Numeric (log ml/min/kg)
	Renal OCT2 substrate	No	Categorical (Yes/No)
Toxicity	AMES toxicity	No	Categorical (Yes/No)
	Max. tolerated dose	0.069	Numeric (log mg/kg/day)
	hERG I inhibitor	No	Categorical (Yes/No)
	hERG II inhibitor	No	Categorical (Yes/No)

	Oral Rat Acute Toxicity (LD50)	2.117	Numeric (mol/kg)
	Oral Rat Chronic Toxicity (LOAEL)	1.131	Numeric (log mg/kg_bw/day)
	Hepatotoxicity	No	Categorical (Yes/No)
	Skin Sensitisation	No	Categorical (Yes/No)
	T.Pyriformis toxicity	0.46	Numeric (log ug/L)
	Minnow toxicity	0.313	Numeric (log mM)

Table S5. List of intercept targets between compound and non-small cell lung cancer cell targets by Venny

EGFR	FLT3	AHR	ALOX12
MET	ESR2	ODC1	XDH
TERT	NOS2	HSD17B1	ALOX15
KIT	TOP2A	INSR	ST6GAL1
PIK3CG	CREB1	MYLK	ESRRA
ESR1	GSK3B	AKR1C3	CA2
SRC	CYP19A1	AKR1C1	P4HB
PIK3R1	IKBKB	MAPT	ADORA2A
AR	MPO	PTPN6	CA12
KDR	APEX1	CSNK2A1	AKR1C2
ABCB1	CYP1B1	PIM1	PDE5A
MMP9	AURKB	PTPN1	CA4
PTGS2	DAPK1	ACHE	BCHE
MMP2	MMP3	ARG1	NUAK1
IGF1R	CA9	NOX4	PLA2G1B
DRD4	ELAVL1	TNKS	ELAVL3
ABCC1	ALOX5	LRP6	NEK6
PTK2	LCK	CBR1	ADORA1
ABCG2	NTRK2	CBS	GRK6
CDK6	PLG	MPG	CA1
MCL1	CXCR1	NEK2	CA14
CDK1	MMP13	ACP1	ALOX12

Figure S4 Intercept targets between compound's targets and non-small cell lung cancer's targets by Venny

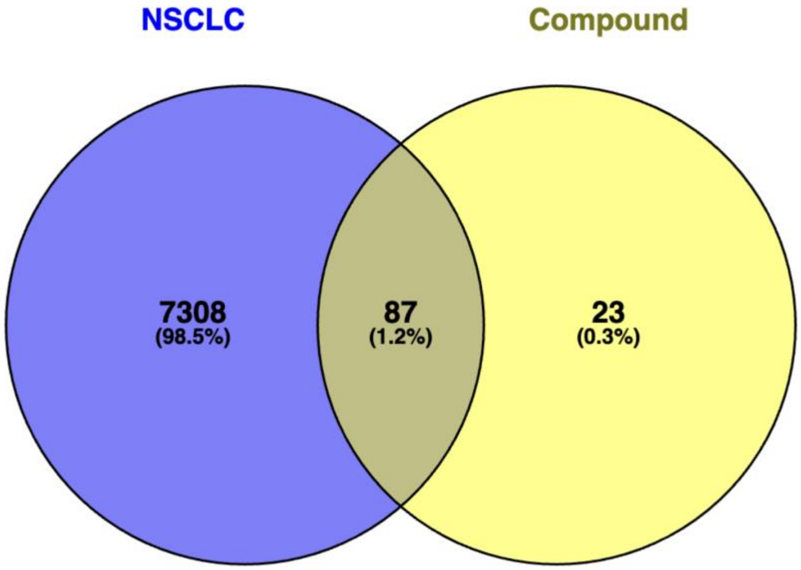
NSCLC
Compound
7308
(98.5%)
87
(1.2%)
23
(0.3%)

Figure S5. Gene Ontology (GO) enrichment analysis based on biological process, molecular function, and cellular component. The data are ranged from the highest significant on the top to those of lower at the bottom.

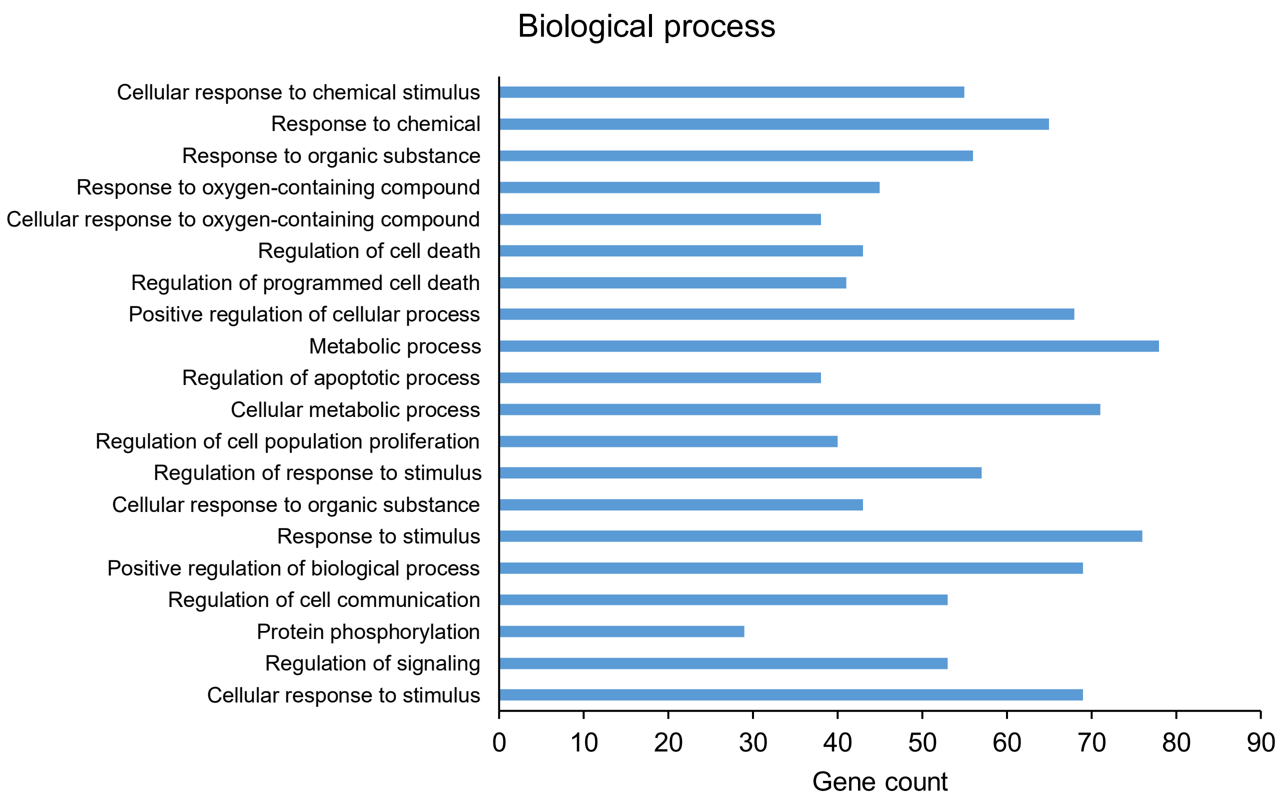

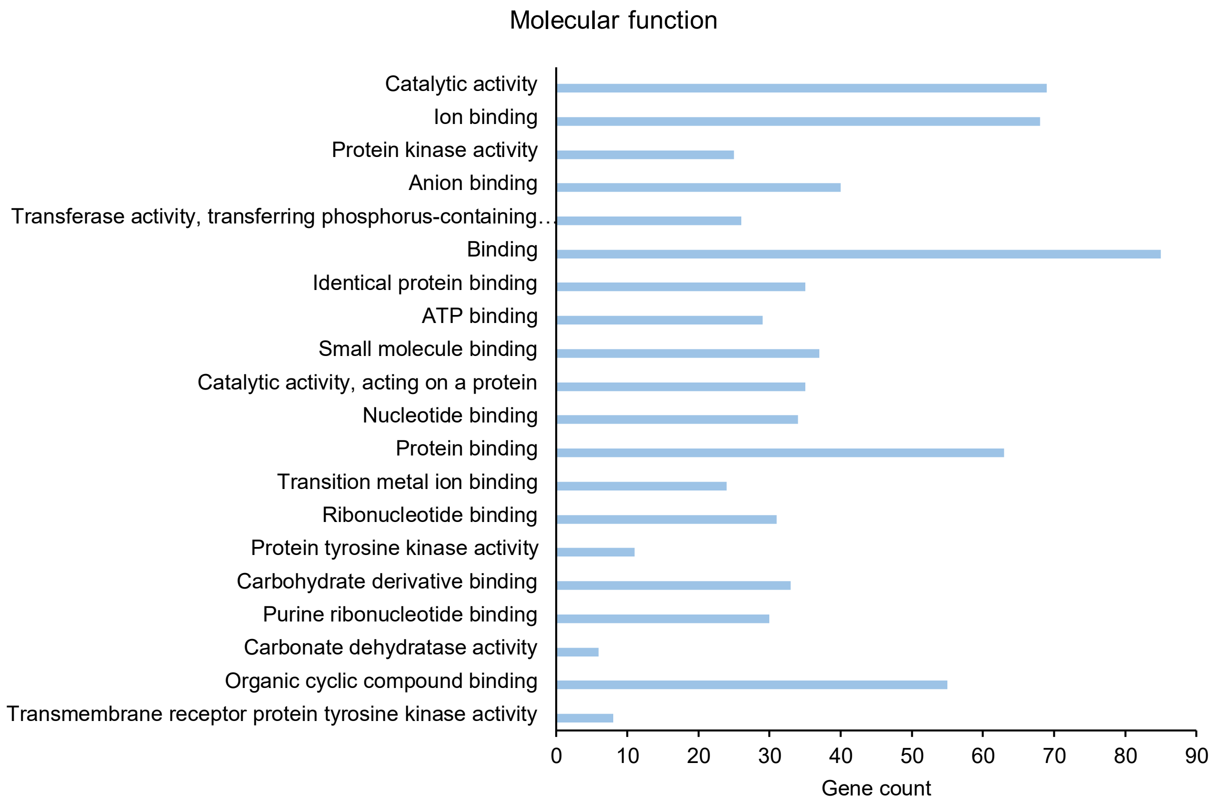

Molecular function
Catalytic activity
Ion binding
Protein kinase activity
Anion binding
Transferase activity, transferring phosphorus-containing…
Binding
Identical protein binding
ATP binding
Small molecule binding
Catalytic activity, acting on a protein
Nucleotide binding
Protein binding
Transition metal ion binding
Ribonucleotide binding
Protein tyrosine kinase activity
Carbohydrate derivative binding
Purine ribonucleotide binding
Carbonate dehydratase activity
Organic cyclic compound binding
Transmembrane receptor protein tyrosine kinase activity
0
10
20
30
40
50
60
70
80
90
Gene count

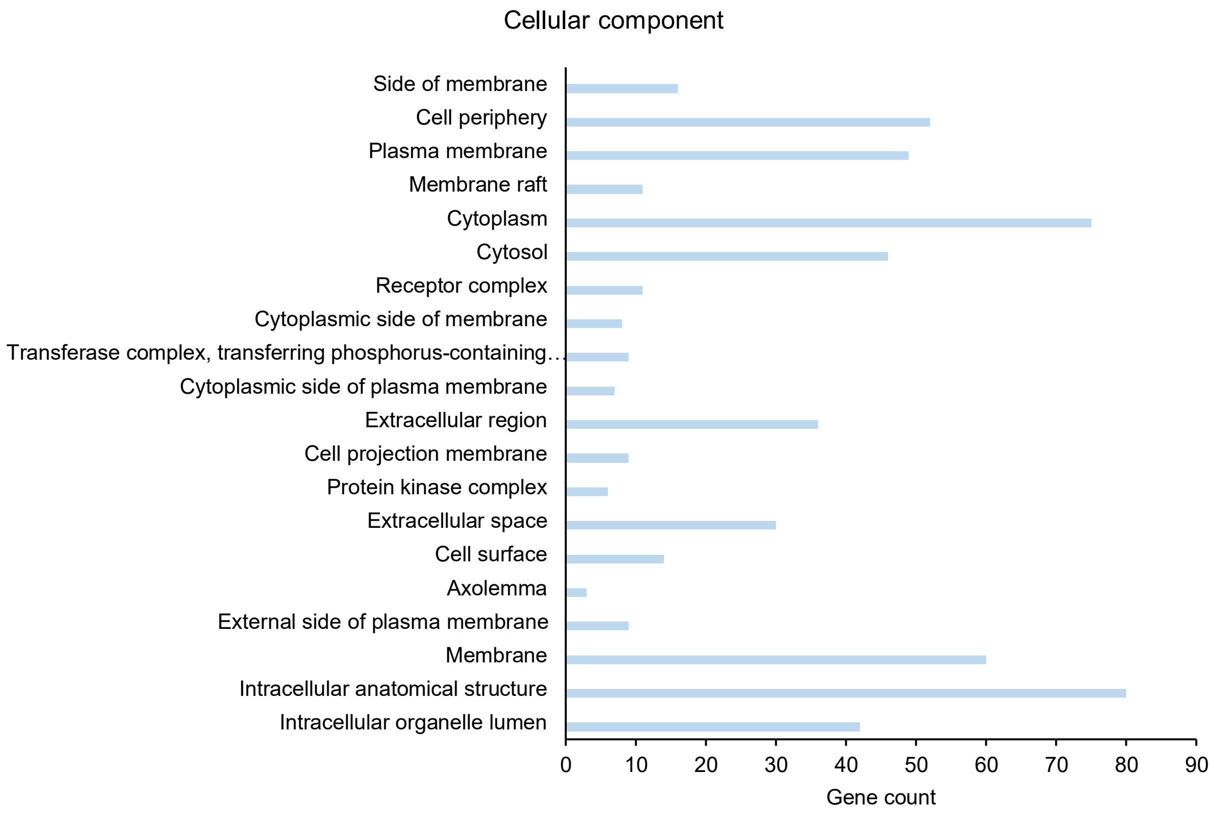

Cellular component
Side of membrane
Cell periphery
Plasma membrane
Membrane raft
Cytoplasm
Cytosol
Receptor complex
Cytoplasmic side of membrane
Transferase complex, transferring phosphorus-containing…
Cytoplasmic side of plasma membrane
Extracellular region
Cell projection membrane
Protein kinase complex
Extracellular space
Cell surface
Axolemma
External side of plasma membrane
Membrane
Intracellular anatomical structure
Intracellular organelle lumen
0 10 20 30 40 50 60 70 80 90
Gene count

Figure S6. KEGG pathway analysis. The data are ranged from the highest significant on the top to those of lower at the bottom.

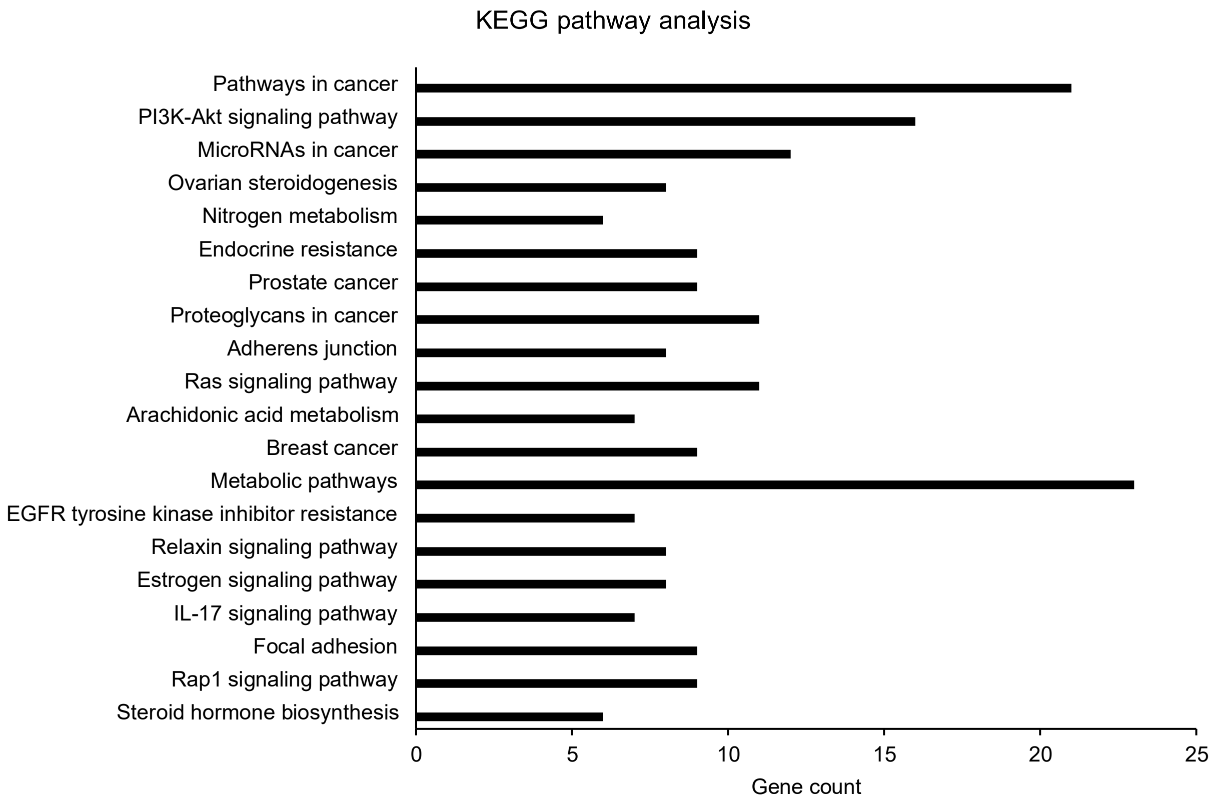

Table S6. Important nodes in network analyzer

Name	Degree	Betweenness Centrality	Closeness Centrality	Clustering Coefficient
SRC	15	0.44474548	0.54716981	0.23809524
PIK3R1	12	0.24312397	0.5	0.27272727
ESR1	8	0.3499179	0.51785714	0.32142857
EGFR	8	0.03493432	0.46774194	0.57142857
IGF1R	7	0.03294335	0.46031746	0.61904762
PTK2	7	0.0091954	0.4084507	0.57142857
AKR1C3	6	0.22660099	0.32222222	0.2
PTPN1	6	0.01297209	0.38666667	0.46666667
PTPN6	5	0.01389573	0.39726027	0.6
KDR	5	0.00402299	0.39726027	0.6
CYP19A1	4	0.33990148	0.4084507	0.33333333

PTGS2	4	0.6	0.66666667	0.5
MET	4	4.93E-04	0.37662338	0.83333333
MMP9	4	0.13546798	0.38157895	0.16666667
HSD17B1	3	0.02955665	0.31182796	0.66666667
ALOX12	3	0	0.5	1
ALOX15	3	0	0.5	1
ALOX5	3	0	0.5	1
TOP2A	3	0.16666667	1	0.66666667
CDK1	3	0.16666667	1	0.66666667
GSK3B	3	1	1	0
ESR2	3	0.08103448	0.44615385	0.66666667
INSR	3	0.00178571	0.35365854	0.66666667
PIK3CG	3	0	0.38666667	1

Figure S7. The top targets in the PPI network as ranked using the cytoHubba plug in network analyzer. The higher degree value is represented by colors ranging from purple to blue.

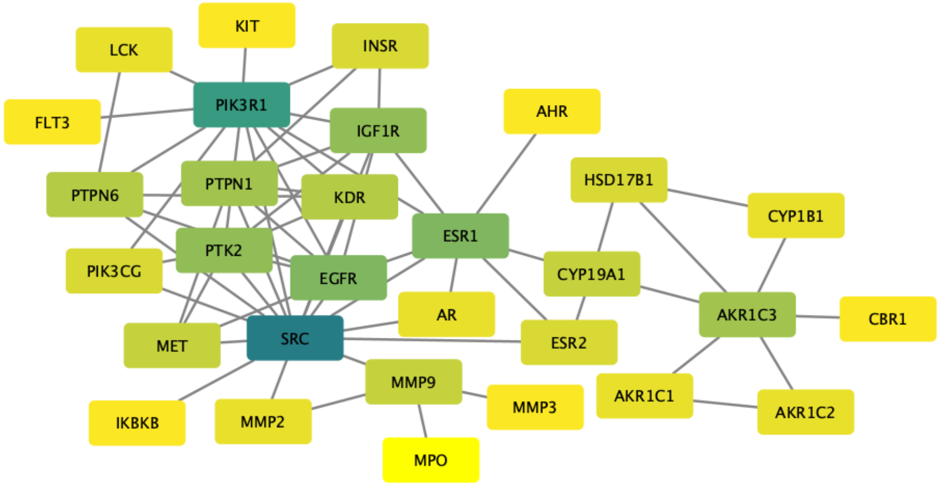
KIT
LCK
INSR
PIK3R1
FLT3
IGF1R
AHR
PTPN6
PTPN1
KDR
HSD17B1
CYP1B1
ESR1
PTK2
PIK3CG
EGFR
CYP19A1
AR
AKR1C3
CBR1
MET
SRC
ESR2
MMP9
AKR1C1
MMP3
AKR1C2
IKBKB
MMP2
MPO

Docking result: activated Akt/1O6L with Isalpinin

Ligand: COC1=CC(O)=C2C(=O)C(O)=C(OC2=C1)C1=CC=CC=C1

Table S7: CurPocket-based information on the five largest binding pockets of pAkt protein.

Binding Pocket	Cavity Volume ($Å^3$)	Center (x, y, z)	Cavity size (x, y, z)
C1	1857	43, 30, 107	23, 16, 25
C2	386	23, 24, 123	8, 19, 15
C3	239	47, 46, 113	9, 11, 11
C4	230	45, 43, 135	9, 9, 6
C5	200	23, 28, 111	8, 13, 7

Table S8: Binding pockets with the greatest negative Vina scores after blind docking with Isalpinin.

Binding pocket	Vina Score (kcal/mol)	Cavity Volume ($Å^3$)	Center (x, y, z)	Docking size (x, y, z)
C1	-8.2	1857	43, 30, 107	28, 21, 30
C2	-7.1	386	23, 24, 123	21, 21, 21
C4	-6.2	230	45, 43, 135	21, 21, 21
C3	-6.1	239	47, 46, 113	21, 21, 21
C5	-5.1	200	23, 28, 111	21, 21, 21

a

b

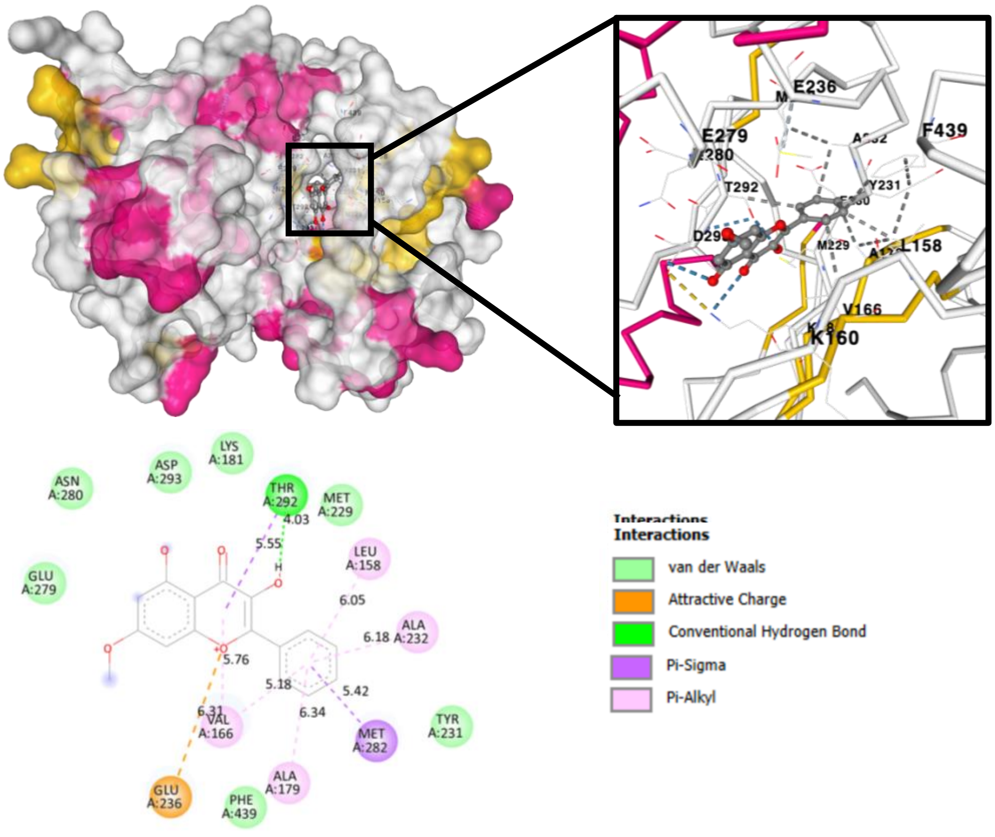

Figure S8. Isalpinin directly binds to activated Akt. (**a**) Molecular docking analysis of the binding Pocket C1 of activated Akt and Isalpinin was performed by CB-Dock2. (**b**) The binding pose, contact residues, and molecular interactions were visualized using BIOVIA discovery studio.
